# *De Novo* Transcriptome Analysis Reveals Abundant Gonad-specific Genes in the Ovary and Testis of *Henosepilachna vigintioctopunctata*

**DOI:** 10.3390/ijms20174084

**Published:** 2019-08-21

**Authors:** Wei Guo, Jing Lü, Mujuan Guo, Shimin Chen, Baoli Qiu, Wen Sang, Chunxiao Yang, Youjun Zhang, Huipeng Pan

**Affiliations:** 1 Key Laboratory of Bio-Pesticide Innovation and Application of Guangdong Province, Engineering Technology Research Center of Agricultural Pest Biocontrol of Guangdong Province, South China Agricultural University, Guangzhou 510642, China; 2 State Key Laboratory for Conservation and Utilization of Subtropical Agro-bioresources, South China Agricultural University, Guangzhou 510642, China; 3 Department of Plant Protection, Institute of Vegetables and Flowers, Chinese Academy of Agricultural Sciences, Beijing 100081, China

**Keywords:** *Henosepilachna vigintioctopunctata*, transcriptome annotation, gonads, differential expression, RNA interference

## Abstract

*Henosepilachna vigintioctopunctata* (Coleoptera: Coccinellidae) is a major pest affecting Solanaceae plants in Asian countries. In this study, we sequenced the ovary and testis transcriptomes of *H. vigintioctopunctata* to identify gonad-related genes. Comparison of the unigene sequences in ovary and testis libraries identified 1,421 and 5,315 ovary- and testis-specific genes, respectively. Among the ovary-specific genes, we selected the *RC2-like* and *PSHS-like* genes to investigate the effects of gene silencing on the mortality, percentage infertility, pre-oviposition period, fecundity, daily number of eggs laid, and hatching rate in female adults. Although the percentage mortality and infertility of females did not differ significantly among dsRNA treatments, fecundity was significantly reduced in the dsRC2-like and dsPSHS-like treatment groups. Moreover, the pre-oviposition period was markedly prolonged in response to dsPSHS-like treatment. This is the first reported RNA sequencing of *H. vigintioctopunctata*. The transcriptome sequences and gene expression profiles of the ovary and testis libraries will provide useful information for the identification of gonad-related genes in *H. vigintioctopunctata* and facilitate further research on the reproductive biology of this species. Moreover, the gonad-specific genes identified may represent candidate target genes for inhibiting the population growth of *H. vigintioctopunctata*.

## 1. Introduction

*Henosepilachna vigintioctopunctata* (Fabricius) (Coleoptera: Coccinellidae) is a widely distributed beetle in Asian countries, including China, India, Japan, and Korea [1,2,3,4]. This species is considered to be a phytophagous pest that mainly causes damage to Solanaceae plants, particularly eggplants, along with other crops such as potatoes, pumpkins, and rock melons [5]. Both the adult and larval stages of *H. vigintioctopunctata* feed on the leaves of these plants by scraping the leaf cuticle, thereby promoting the development of irregular transparent spots or perforations in the injured leaves, which later become brown depression marks. In addition, these beetles can cause notable damage to tender stems, petals, sepals, and fruits, resulting in reduced plant yield, poor fruit quality, or plant withering [6,7]. During the past 20 years, a large number of studies have been conducted on the basic ecology and biology of *H. vigintioctopunctata* [8,9,10,11,12]. At present, chemical pesticides are primarily used for controlling *H. vigintioctopunctata*. However, given the current requirement for negative growth in pesticide use in China, as stipulated in the 2019 “No. 1 Central Document,” there is an urgent need to develop effective new pest control methods that target *H. vigintioctopunctata* based on novel modes of action.

In this latter regard, RNA interference (RNAi) provides an effective molecular tool for the determination of gene function, and can also be exploited to facilitate the development of targeted and environmentally friendly pest control methods with new modes of action [13,14,15,16,17,18]. In terms of pest control, RNAi-mediated silencing has shown excellent efficacy and has been applied in the control of a number of coleopteran insects, including the Western corn rootworm *Diabrotica virgifera virgifera* [19], red flour beetle *Tribolium castaneum* [20], and the Colorado potato beetle *Leptinotarsa decemlineata* [20,21]. Moreover, our preliminary experimental results indicated that dietary RNAi targeting multiple genes is particularly effective against *H. vigintioctopunctata*, indicating that RNAi has potential utility in the control of this species.

From the perspective of pest control, gaining an understanding of the genes involved in gonadal differentiation and reproduction could make a considerable contribution toward the development of novel control strategies. Numerous genes have been investigated to evaluate their roles in the gonadal differentiation and reproduction of insects, including *transformer 2* in the Asian citrus psyllid *Diaphorina citri* [22]; *vATPase A*, *vATPase E*, *vitellogenin*, and *Brahma* in the common bed bug *Cimex lectularius* [23,24,25]; *lipase maturation factor* in the grain aphid *Sitobion avenae* [26]; *vitellogenin* receptor and *boule* in *D. virgifera virgifera* [27]; *Ran* in the brown planthopper, *Nilaparvata lugens* [28]; *Fatty acyl-Co A reductase* in the cotton plant bug *Adelphocoris suturalis* [29]; and *boule*, *Zero growth Population*, *double sex male*, *fzo,* and *gas8* in the fruit fly *Bactrocera dorsalis* [30]. Silencing these genes that are highly expressed in the ovary or testis has been shown to result in markedly reduced egg production and a lower rate of hatching. Although the egg production capacity of *H. vigintioctopunctata* is relatively high, with each female capable of laying approximately 618 eggs in its lifetime [9], the regulatory molecular aspects of reproduction in *H. vigintioctopunctata* remain unclear. Accordingly, we reasoned that a molecular investigation of *H. vigintioctopunctata* reproduction would provide important insights with regard to identifying novel target sites for population control.

The first step on the road to understanding the molecular mechanisms of reproduction is to identify gonad-related genes. In this study, we employed Illumina-based sequencing and *de novo* assembly to screen for gonad-related genes by constructing the transcriptomes of ovaries and testes in *H. vigintioctopunctata*. We accordingly obtained 69,854 unigenes from the six libraries comprising the ovary and testis transcriptomes of *H. vigintioctopunctata*. Moreover, we performed comparative transcriptome analysis to identify the genes that were differentially expressed between ovaries and testes, among which, 13 and 16 candidate unigenes that were upregulated in the ovary and testis, respectively, were selected to confirm their altered expression levels via RT-qPCR and semi-RT-qPCR analyses. Finally, we investigated the effects of silencing two ovary-specific genes, *RC2-like* and *PSHS-like*, on the mortality, percentage infertility, pre-oviposition period, and fecundity of adult females, along with the number of eggs laid daily and egg hatching rate. By comparing unigene sequences in the ovary and testis libraries, we were able to determine gonad-specific differentially expressed genes, which will contribute to a more comprehensive understanding of the regulatory mechanisms involved in gonadal differentiation and reproductive processes in *H. vigintioctopunctata*. Furthermore, these gonad-specific genes can potentially be used as targets for suppressing the population growth of this species.

## 2. Results

### 2.1. Illumina Sequencing and Assembly

To identify gonad-specific/differentially expressed genes in *H. vigintioctopunctata*, we sequenced cDNA libraries from ovaries and testes using an Illumina HiSeqTM 2500 sequencer. In total, 270,948,088 bp of raw reads were generated from six *H. vigintioctopunctata* ovary and testis libraries. Following quality control, 269,276,962 bp of clean reads were retrieved after removing low-quality reads and trimming adapters. Details of the sequencing and subsequent assembly are shown in Table 1.

**Figure 1:**
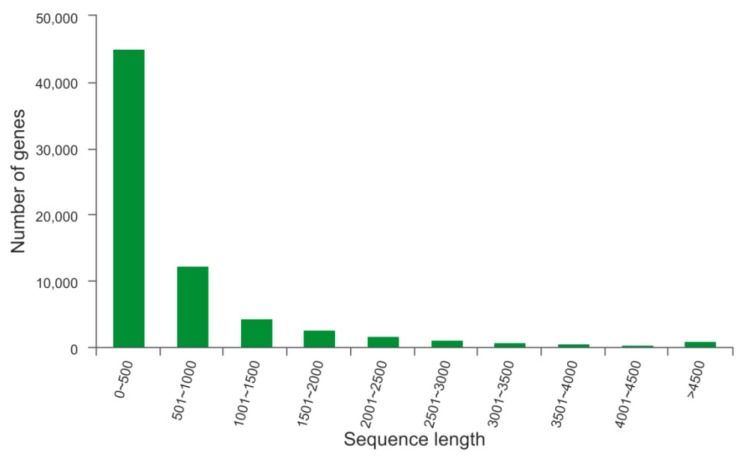
Sequence length distribution of all unigenes. X-axis, sequence length of unigenes. Y-axis, the number of unigenes in different length ranges. Sixty-five percent of reads were ≤ 500 bp in size.

*De novo* assembly of the clean reads generated 69,854 unigenes with an N50 of 1284 bp and an average length of 780 bp (Table 1). The sequence length distribution of the assembled unigenes is shown in Figure 1. We found that the unigenes were primarily concentrated in the 0–500 bp range (65%), followed by unigenes between 501 and 1000 bp in length (18%).

### 2.2. Annotation and Functional Classification

**Figure 2:**
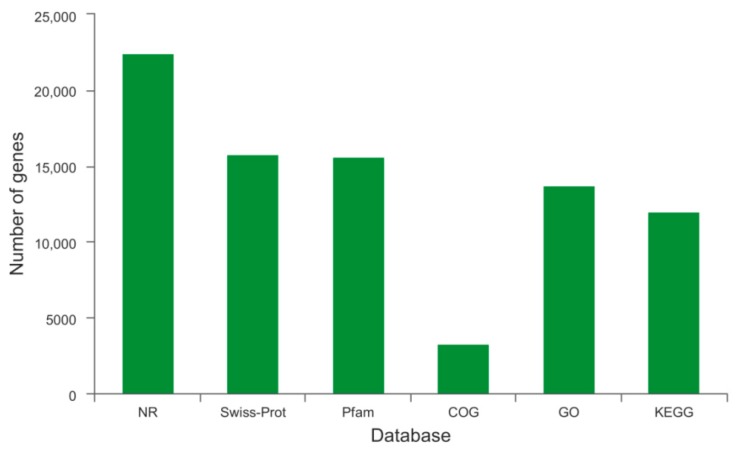
Statistics of annotation results.

In total, 69,854 unigenes were subjected to annotation analysis by matching sequences against six commonly used databases: Nr, Swiss-Prot, Pfam, COG, GO, and KEGG (Figure 2). Among these unigenes, 22,472 (32.17% of the total), 15,765 (22.57%), 15,617 (22.36%), 3,261 (4.67%), 13,702 (19.62%), and 11,970 (17.14%) were matched with sequences in the Nr, Swiss-Prot, Pfam, COG, GO, and KEGG databases, respectively (Table 2).

**Figure 3:**
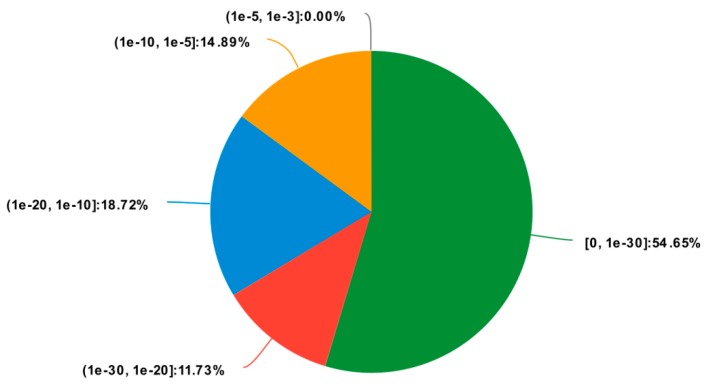
Distribution of the e-values for annotated unigenes.

The 22,472 annotated unigenes matched known sequences from different species. The majority of unigene hits were to the red flour beetle *T. castaneum* (22.73%), the Asian long-horned beetle *Anoplophora glabripennis* (15.65%), the small hive beetle *Aethina tumida* (7.42%), the Colorado potato beetle *L. decemlineata* (6.52%), the taurus scarab *Onthophagus taurus* (2.40%), the drywood termite *Cryptotermes secundus* (2.03%), and the mountain pine beetle *Dendroctonus ponderosae* (2.00%) (Table 3). In total, 12,282 (54.65%) unigenes shared effective similarities at an e-value of e ≤ 1e-30, 2,636 (11.73%) shared effective similarities at an e-value of 1e^−30^ to 1e^−20^, and 4,207 (18.72%) shared significant similarities at an e-value of 1e^−20^ to 1e^−10^. The remaining 3,347 (14.89%) unigenes shared a low similarity at an e-value from 1e^−10^ to 1e^−5^. The distribution of the e-values for the annotated unigene sequences is shown in Figure 3.

In total, 22,472 unigenes shared effective similarity with the matched sequences (e ≤ 1e^−5^).

### 2.3. Gene Ontology Analysis

**Figure 4:**
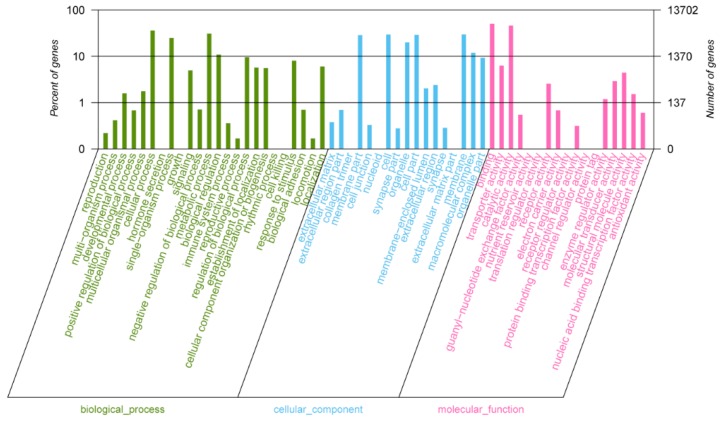
Distribution of the annotated unigenes in three GO categories (level 2).

Totals of 18,823, 22,538, and 16,126 unigenes were assigned to the three GO categories biological process, cellular component, and molecular function, respectively (Figure 4). In the biological process category, the level 2 terms cellular process (25.51%), metabolic process (21.52%), and single-organism process (14.90%) were the most frequently represented, whereas in the cellular component category, the level 2 terms cell (18.00%), membrane (17.93%), cell part (17.67%), and membrane part (17.30%) were the predominant categories, and in the molecular function category, binding (42.83%) and catalytic activity (39.14%) were the most abundant.

### 2.4. KEGG Analysis

**Figure 5:**
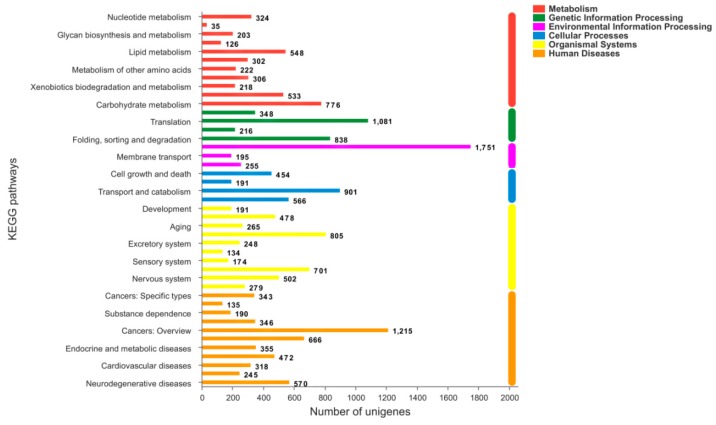
Distribution of the mapped KEGG pathways. X-axis: the number of unigenes mapped to each KEGG pathway. Y-axis: distribution of KEGG pathways.

We specifically assigned 11,970 unigene sequences to 307 KEGG pathways, the most abundantly represented of which were ubiquitin-mediated proteolysis, RNA transport, cAMP signaling pathway, purine metabolism, microRNAs in cancer, pathways in cancer, and others (Figure 5).

### 2.5. Analysis of Differentially Expressed Genes in Ovary and Testis

**Figure 6:**
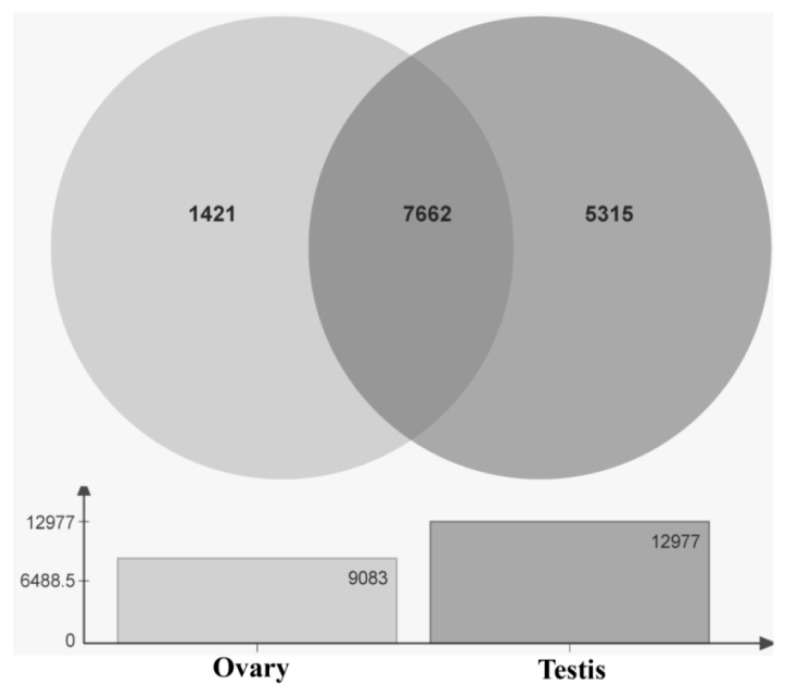
The total number of unigenes and their expression in the ovary and testis libraries.

**Figure 7:**
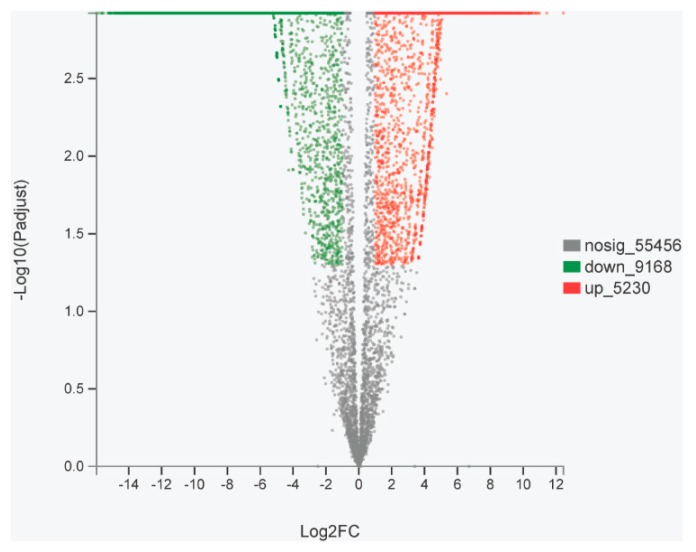
Volcano plot of differences in gene expression between ovaries and testes. Upregulated represents ovary-biased genes, whereas downregulated represents testis-biased genes.

On the basis of a comparison of the unigenes in the ovary and testis transcriptomes, we identified 1,421 and 5,315 genes that are specifically expressed in the ovaries and testes, respectively. Furthermore, we found that 7,662 unigenes are expressed in both the ovaries and testes (Figure 6). In addition, compared with the testes, 5,230 and 9,168 differentially expressed genes were upregulated and downregulated, respectively, in the ovaries (Figure 7). Using annotation information on the differentially expressed genes in ovary and testis, we screened and identified 438 functional genes related to gonadal development processes in the ovary and testis transcriptomes (Table S1).

### 2.6. Discovery of Molecular Markers

**Figure 8:**
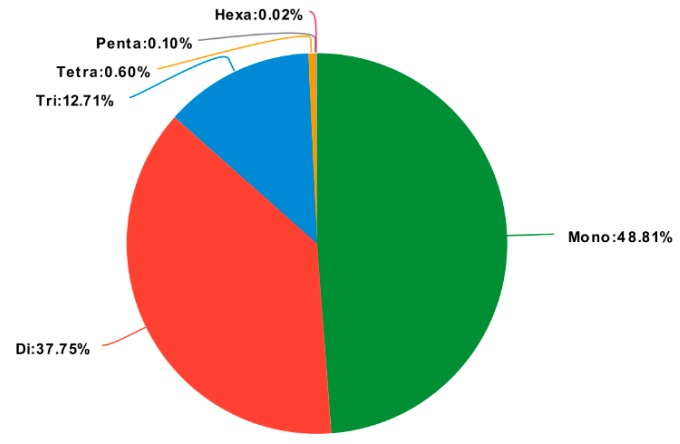
Distribution of identified SSRs according to motif type.

We detected 4,964 SSRs in our six transcriptome libraries (Figure 8), among which, the largest number of SSR motifs were mono-nucleotide repeats (2,423), accounting for 48.81% of all the predicated SSRs, followed by di-nucleotide (37.75%), tri-nucleotide (12.71%), tetra-nucleotide (0.60%), penta-nucleotide (0.10%), and hexa-nucleotide (0.02%) repeats. In addition, among the 4,213 unigenes containing SSRs, 303 unigenes were predicated to contain more than one SSR (Table 4).

### 2.7. Semi-RT-qPCR and RT-qPCR Confirmation of Gonad-biased Genes

**Figure 9:**
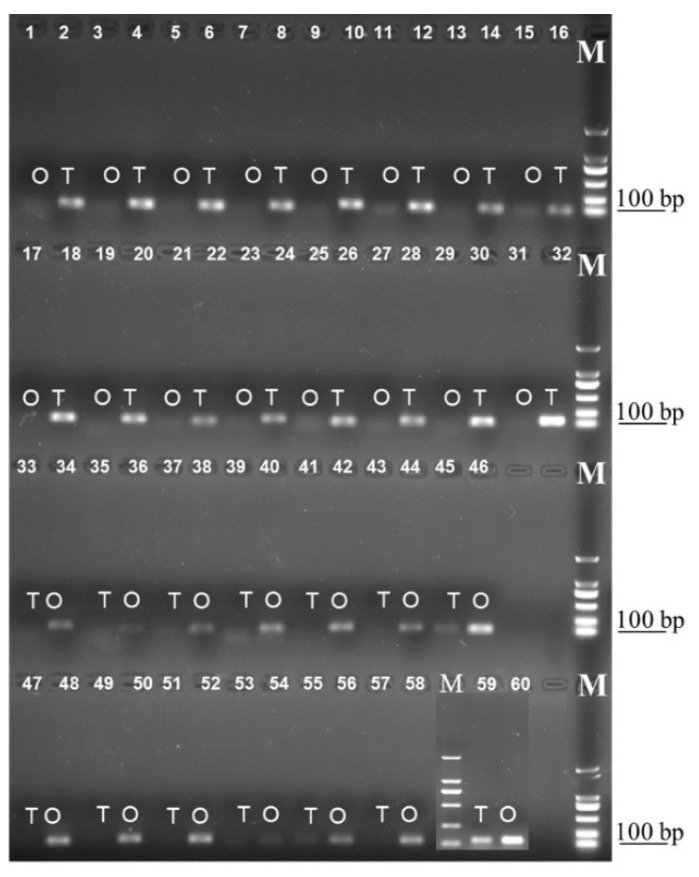
Validation of gonad differentially expressed genes. T, testis; O, ovary; M, marker. (1–32) Testis up-regulated genes; (33–58) Ovary up-regulated genes; (59–60) *RPS18*. Details of the genes shown in the figure are presented in Table S3.

In total, 13 ovary-upregulated and 16 testis-upregulated unigenes were selected to verify their expression profiles using semi-RT-qPCR and RT-qPCR. The semi-RT-qPCR and RT-qPCR results were found to be consistent with those obtained from RNA-seq analysis (Figure 9, Tables S2 and S3). Among these genes, protein D3-like and ceramide-1-phosphate transfer protein were expressed in both the ovary and testis, whereas the other genes were expressed exclusively in either the ovary or testis (Table S2). However, we found that the two commonly expressed genes showed tissue-specific expression patterns. Specifically, the protein D3-like unigene was highly expressed in the testis, whereas expression levels were lower in the ovary. Conversely, the expression of the ceramide-1-phosphate transfer protein unigene was found to be considerably higher in the ovary than in the testis.

### 2.8. The Effects of Dietary RNAi of dsPSHS-like and dsRC2-like on PSHS-like and RC2-like Gene Expression

**Figure 10:**
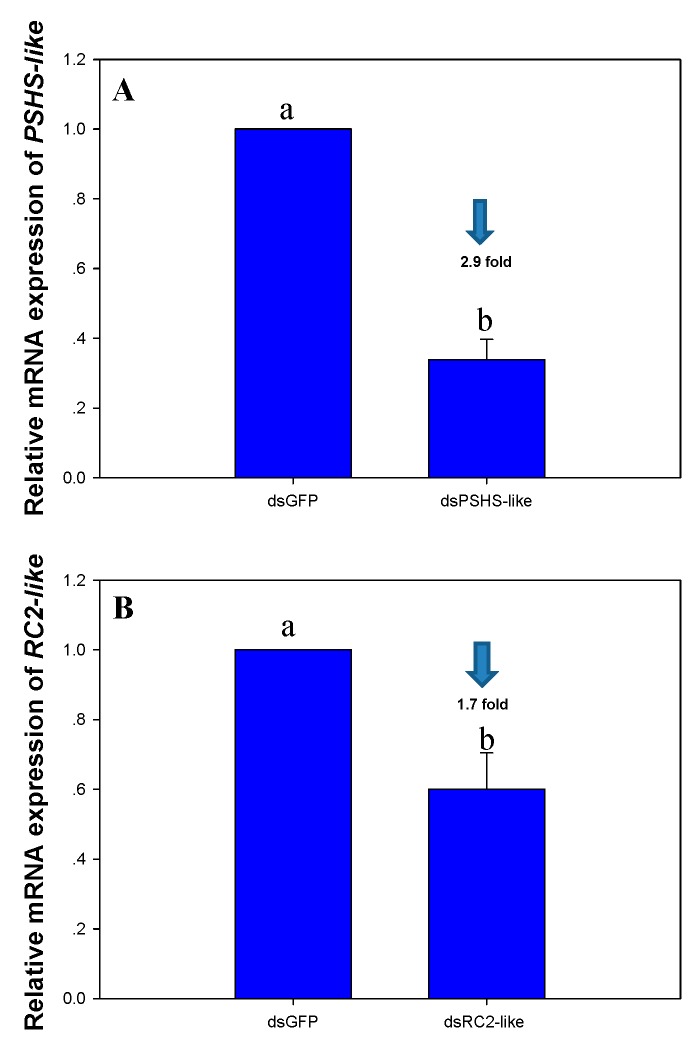
Changes in the expression of *PSHS-like* and *RC2-like* genes in the ovary of *Henosepilachna vigintioctopunctata* after ingestion of dsPSHS-like or dsRC2-like compared with dsGFP. The values displayed are the means + SE. Different letters indicate significant differences between treatments (*p* < 0.05).

The results of RNAi analysis showed that expression of the *PSHS-like* and *RC2-like* genes in the ovary under the dsPSHS-like (F_1, 8_ = 128.479, *p* < 0.0001) and dsRC2-like (F_1, 8_ = 14.616, *p* = 0.005) treatments was significantly suppressed compared with those in the dsGFP control group. Specifically, the relative expression levels of *PSHS-like* and *RC2-like* were suppressed by 2.9-fold and 1.7-fold, respectively, compared with those of the dsGFP control group (Figure 10).

### 2.9. The Effects of Silencing dsPSHS-like and dsRC2-like on the Phenotype of H. vigintioctopunctata

**Figure 11:**
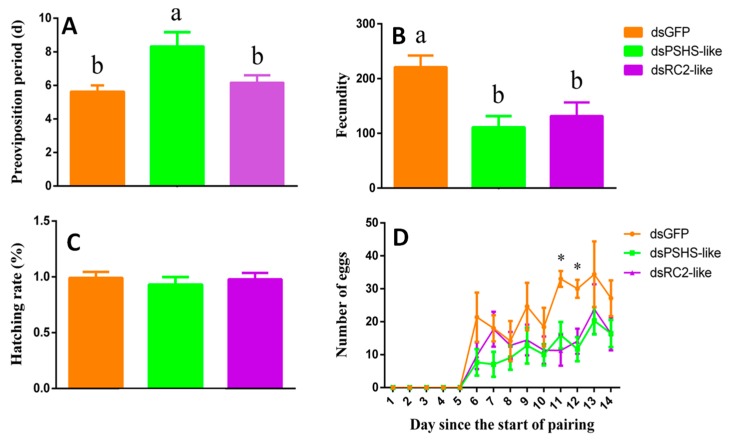
Phenotypic effects of silencing *RC2-like* and *PSHS-like* using in vitro-synthesized dsRNAs. (**A**) Pre-oviposition period. (**B**) Fecundity. (**C**) Hatching rate. (**D**) Number of eggs laid each day. The values shown are the means ± SE. Within each panel, different letters indicate significant differences between treatments (*p* < 0.05). In panel D, ‘*’ indicates a significant difference among treatments on the 11th and 12th day (*p* < 0.05).

We investigated the effects of silencing *RC2-like* and *PSHS*-*like* on mortality, percentage infertility, pre-oviposition period, fecundity, number of eggs laid daily, and hatching rate by feeding dsRNAs to the adult females of *H. vigintioctopunctata* and subsequently observing the treated individuals for 14 days. Whereas we observed zero female mortality in the dsGFP control group, 10.53% and 26.32% of females died in the dsPSHS-like and dsRC2-like-treatment groups, respectively. Furthermore, apart from a single female in the dsRC2-like-treatment group, which died after laying eggs for 2, days. all the other females that died did so without laying eggs. Chi-square tests showed that there was no significant difference in mortality among the dsRNA treatments (χ^2^ = 4.538, *p* = 0.103). Additionally, among the paired females in the dsGFP, dsPSHS-like, and dsRC2-like groups, 33.33%, 26.32%, and 21.05%, respectively, did not lay eggs, although the percentage infertility did not differ significantly among the treatments (χ^2^ = 0.578, *p* = 0.749). Furthermore, we found that the pre-oviposition period was markedly prolonged under the dsPSHS-like treatment compared with that in the dsRC2-like and dsGFP groups (F_2, 28_ = 5.045, *p* = 0.013) (Figure 11A), and total fecundity was significantly reduced in the dsRC2-like and dsPSHS-like treatment groups compared with the dsGFP control (F_2, 28_ = 5.820, *p* = 0.008) (Figure 11B). Moreover, the number of eggs laid on the 11th and 12th day in the dsGFP control group significantly exceeded that in the dsPSHS-like and dsRC2-like treatment groups (Figure 11D). In contrast, the rate of egg hatching did not vary significantly among the treatment and control groups (F_2, 28_ = 0.265, *p* = 0.769) (Figure 11C).

## 3. Discussion

Although *H. vigintioctopunctata* has become an increasingly serious pest of solanaceous and cucurbitaceous plants in China in recent years, there is still relatively little genomic information available for this beetle. In this regard, transcriptome analysis can provide a basis for future gene expression and functional analyses of *H. vigintioctopunctata*. The findings of the present study will also augment the limited sequence data currently available for phytophagous ladybugs.

In this study, we performed RNA-seq analysis of the ovaries and testes of newly eclosed adult males and females of *H. vigintioctopunctata* in an attempt to identify gonad-related genes. We accordingly obtained 69,854 unigenes with an average length of 780 bp. This length is considerably longer than the unigenes obtained from the gut and salivary glands of the whitefly *Bemisia tabaci* [31,32] and the intestines and fat bodies of the brown planthopper *N. lugens* [33,34], although notably shorter than the unigenes from the small white butterfly *Pieris rapae* [35]. The 69,854 unigenes with the highest number of BLASTx hits with the sequences of other species revealed high homology with several other coleopteran species, for which complete genome sequences are currently available. Nevertheless, we succeeded in annotating only 22,472 (32.17%) of the unigenes obtained, possibly owing to the limited published data for phytophagous ladybug transcriptomes. Accordingly, further research is necessary to functionally characterize these genes.

Gene ontology distributions are known to differ substantially, both among the transcriptomes of various body parts within the same insect and between the transcriptomes of different insects [36,37,38]. For example, the GO distribution determined in the present study was found to show a pattern similar to the gonad transcriptomes of other insects, such as the testis transcriptomes from the cockroach *Periplaneta americana* [36] and the oriental fruit fly *Bactrocera dorsalis* [38]. However, it differs significantly from the transcriptomes of many other insects, such as the antennal transcriptome of the Asian long-horned beetle *A. glabripennis* [37] and the intestine-specific transcriptome of the brown planthopper *N. lugens* [33]. We believe that the genes that are highly or specifically expressed in gonads show marked difference from those in other tissues because sex-biased expression tends to be high in the gonads but lower in other tissues [39,40,41,42]. This should perhaps be expected, given that the gonads show considerably greater sexual dimorphism than other tissues.

Comparisons of testis and ovary transcriptomes have previously been conducted for the green mud crab *Scylla paramamosain* [43], the swimming crab *Portunus trituberculatus* [44], and the Pacific white shrimp *Litopenaeus vannamei* [45]. Similarly, in the present study, we specifically sought to identify the gonad specifically/differentially expressed genes in *H. vigintioctopunctata*. To the best of our knowledge, this is the first study to compare the testis- and ovary-specific transcriptomes of the same insect. Indeed, to date, the testes transcriptomes of only a relatively few insect species have been sequenced [36,38]. Among those that have been sequenced, 125,390 unigenes were obtained from the testis transcriptome of *P. americana* [36], whereas only 20,921 unigenes were annotated from the testes of *B. dorsalis* [38]. Therefore, we had very little data to compare with the number of testis- and ovary-specific genes identified in *H. vigintioctopunctata*. The 1,421 and 5,315 genes identified as being specifically expressed in the ovary and testis of *H. vigintioctopunctata* will thus provide important genetic information for the study of reproductive regulatory mechanisms in this species. Our preliminary study showed that, compared with the reproductive system of unmated females (Figure S1B), that in the mated females in the dsGFP control group developed normally (Figure S1A). In contrast, we found that the reproductive system of the mated females that did not lay eggs had developed abnormally (Figure S1C). Furthermore, we noted that there was no obvious difference among the reproductive systems of the egg-laying mated females in the dsGFP control group and the dsRC2-like and dsPSHS-like treatment groups. However, although a large number of genes related to gonadal development have been characterized in the ovary and testis transcriptomes of *H. vigintioctopunctata* (Table S1), the precise role of these genes in the gonadal development and differentiation processes of this species remain to be determined.

In terms of quantity, the number of testis-specific unigenes identified was substantially greater than that of ovary-specific genes, which could be attributable to the fact that the ovaries and testis were dissected from the newly emerged *H. vigintioctopunctata* adults, indicating that original female development represents a type of “default” state. In order to validate the gene expression data obtained from transcriptomes analysis, we selected 13 and 16 genes that were specifically upregulated in the ovary and testis, respectively, for semi-RT-qPCR and RT-qPCR analysis using the same cDNA samples. We accordingly found that the expression levels of these 29 unigenes in ovary and testis were consistent with the transcriptome analysis, indicating that the transcriptome data were reliable.

In this study, we also randomly selected two genes, namely, *RC2-like* and *PSHS-like*, that were highly and specifically expressed in the ovary to investigate their roles based on the dietary delivery of dsRNAs. We accordingly recorded 10.53% and 26.32% mortality among the females in the dsPSHS-like and dsRC2-like treatment group, respectively, although this mortality was not significantly different from that of females in the dsGFP control group. Importantly, however, the fecundity of females was significantly reduced in the dsRC2-like and dsPSHS-like treatment groups compared with the dsGFP control females, and the pre-oviposition period was found to be markedly prolonged in the dsPSHS-like-treated females compared with those in the dsRC2-like and dsGFP groups. Therefore, we can speculate that *RC2-like* and *PSHS-like,* particularly the latter, could be used to inhibit the population growth of *H. vigintioctopunctata*.

## 4. Materials and Methods

### 4.1. Insect Rearing and Sample Collection

*H. vigintioctopunctata* were reared on eggplant leaves in the laboratory as described in our previous study [46]. The ovaries and testes were dissected from adult males and females (collected during the first 2 days after emergence). All samples were rapidly frozen in liquid nitrogen and stored at −80 °C. Samples were collected in triplicate and these biological replicates were processed independently.

### 4.2. RNA Isolation, Library Preparation, and Sequencing

Total RNA was extracted using a RNeasy MinElute Cleanup Kit (Qiagen, Hilden, Germany) according to the manufacturer’s instructions. Concentrations of the extracted RNA were determined using a NanoDrop 2000 spectrophotometer (Thermo Scientific, Madison, Wisconsin, USA) and the quality was verified using an Agilent 2100 Bioanalyzer (Agilent Technologies, Palo Alto, California, USA). Ovary- and testis-specific RNA samples were pooled at an equal concentration. cDNA libraries were sequenced by Majorbio (Majorbio, Shanghai, China) using an Illumina platform (Illumina, San Diego, California, USA) with sequence runs of 2 × 150-bp paired-ends. The raw reads have been deposited in the Short Read Archive (SRA) of NCBI with the accession number PRJNA531518.

### 4.3. Sequence Assembly and Functional Annotation

The reads were initially assembled using Trinity (V2.2.0) [47], after which duplicated contigs were removed using cd-hit [48] to yield a unigene sequence file.

These unigenes were subsequently annotated against NR, Swiss-Prot, Pfam, COG, GO, and KEGG databases using associated software, the details and parameters of which are listed in Table 5.

### 4.4. Identification of Differentially Expressed Genes in Ovary and Testis 

In order to compare the unigene sequences of the ovary and testis libraries, we performed BLASTN alignments. The best matched (identity ≥90%) sequences were identified as genes that were similarly expressed in both libraries, whereas those with lower matches (identity <90%) were considered to be differentially expressed between the ovary and testis libraries. The Fisher P test was used to determine the differential expression of each pair of common genes, with a Fisher *p*-value of < 0.05 being considered indicative of a significant difference in gene expression.

### 4.5. Molecular Marker Detection

Simple sequence repeats (SSRs) were detected using MISA software [49]. Six types of SSRs were investigated, namely, mono-, di-, tri-, quad-, penta-, and hexa-nucleotide repeats.

### 4.6. Semi-RT-qPCR and RT-qPCR Confirmation of Gonad-biased Genes

Semi-RT-qPCR and RT-qPCR were used to verify gene expression profiles in testis and ovary, as identified from the Illumina sequencing data. In total, 13 and 16 unigenes showing upregulation in ovaries and testes, respectively, were selected to confirm their altered expression levels using the same RNA samples as used for transcriptome sequencing. The RT-qPCR primers used for amplification are shown in Table S3. Gene expression level was initially examined by semi-RT-qPCR, with the PCR products being analyzed electrophoretically using 1.0% agarose gels. We subsequently conducted RT-qPCR to verify the expression profiles in the ovary and testis. Reaction mixtures consisted of 5.25 µL of ddH_2_O, 7.5 µL of 2× SYBR Green MasterMix (Bio-Rad Laboratories, Hercules, California, USA), 4 μM of each specific primer, and 1.0 µL of first-strand cDNA template. The RT-qPCR program included an initial denaturation for 3 min at 95°C, followed by 40 cycles of denaturation at 95 °C for 10 s, annealing for 30 s at 55 °C, and extension for 30 s at 72 °C. The RT-qPCR reactions were run in triplicate in 96-well format Microseal PCR plates (Bio-Rad Laboratories, Hercules, California, USA) and carried out in a CFX96 Touch™ Real-Time PCR Detection System (Bio-Rad Laboratories, Hercules, California, USA), with the *RPS18* gene being used as internal control [46]. RT-qPCR was performed using SYBR^®^ Premix Ex Taq™ (Tli RNaseH Plus) (TaKaRa, Dalian, China) in 15-µL reaction mixture containing 5 ng cDNA as the template. 

### 4.7. Dietary RNAi Toxicity Assay

We determined the role two ovary-specific genes with the assembly IDs TRINITY_DN37464_c0_g3 (REST corepressor 2-like, *RC2-like*) and TRINITY_DN34758_c0_g1 (protein snail homolog Sna-like, *PSHS-like*) (Tables S2 and S3). Specific dsRNA primers containing a T7 promoter sequence at the 5ʹ end targeting these two genes were designed using E-RNAi [50], the sequences of which are listed in Table S4. After amplification from the cDNA samples of *H. vigintioctopunctata* adults and confirmation by sequencing, the amplicons (394 bp for dsRC2-like and 398 bp for dsPSHS-like) were used as templates for in vitro transcription reactions to generate dsRNAs using a T7 MEGAscript kit (Ambion, Austin, Texas, USA).

For the phenotypic investigations, the sex of the newly emerged adults (<12 h) was determined under a stereoscope, and each individual was thereafter reared independently in a petri dish. The adult males were provided with normal leaves, whereas the adult females were provided with leaf discs treated with dsRNAs for five consecutive days. The leaf discs (12 mm diameter) were pre-treated by immersing in one of three 200 ng/µL solutions of dsRNA, namely, dsGFP control, dsRC2-like, or dsPSHS-like. On the fifth day (the first day of pairing), one untreated male and one treated female were paired as a single replicate in individual petri dishes and provided with fresh untreated leaves. As the dsGFP, dsRC2-like, and dsPSHS-like treatment groups, we used 12, 19, and 19 pairs of respectively treated adults. Over the subsequent 14 days, we recorded adult female mortality, the percentage of sterile females, pre-oviposition period, fecundity, number of eggs laid per day, and hatching rate. The ovaries of unmated females, and mated females treated with dsGFP, dsRC2-like, and dsPSHS-like were dissected to evaluate the effects of dsRNA ingestion on ovarian development.

For the evaluation gene expression, adult females were treated with 200 ng/µL dsGFP, dsRC2-like, or dsPSHS-like dsRNA as described above. After 5 days, the ovary was dissected from each adult female. All samples were flash-frozen in liquid nitrogen and stored at −80 °C in 1.5-mL centrifuge tubes prior to total RNA extraction. Each sample was represented by five biological replicates. The reference gene *RPS18* was used as the internal control [46]. Relative quantification of *RC2-like* and *PSHS-like* in the various samples was performed using the 2*^−ΔΔCt^* method [51].

### 4.8. Data Analysis

Student’s *t*-test was used to determine the *RC2-like* and *PSHS-like* expression levels under different treatments. For each comparison, *p* < 0.05 was considered to indicate a significant difference. One-way ANOVA was used to compare data relating to the pre-oviposition period, fecundity, and hatching rate under different treatments. Means were compared using Tukey’s test with the significance set at *p* < 0.05. Proportional data were arcsine square root-transformed prior to analysis. Mortality rates and percentages of sterile females were analyzed using Chi-squared tests. SPSS v. 21.0 (IBM Corp., Armonk, NY, USA) was used for all statistical analyses. 

## 5. Conclusions

To our knowledge, this is the first study in which the RNA sequencing of *H. vigintioctopunctata* has been conducted. As a consequence of sequencing, we obtained more than 269,276,962 clean reads. We identified numerous gonad-related functional genes and accordingly believe that the sequenced transcriptomes will provide useful information for elucidating the molecular mechanisms governing reproduction in *H. vigintioctopunctata*, as well as contributing a source of reference information for other phytophagous ladybugs. Furthermore, we demonstrated the effectiveness of RNAi-based gene silencing in *H. vigintioctopunctata* as a powerful reverse genetics tool for the functional annotation of its genes. Moreover, we showed that gonad-specific genes may represent candidate target genes for inhibiting the population growth of *H. vigintioctopunctata*.

## Figures and Tables

**Table 1:** Statistics of *Henosepilachna vigintioctopunctata* gonad transcriptome sequencing and assembly.

**Raw Results**	
Number of ovary raw reads	133,674,920
Number of testis raw reads	137,273,168
Number of total raw reads	270,948,088
Number of ovary clean reads	132,895,862
Number of testis clean reads	136,381,100
Number of total clean reads	269,276,962
**Assembly results**	
Number of transcripts	99,718
Average length of transcripts (bp)	780
Minimum transcript size (bp)	201
Maximum transcript size (bp)	16,278
N50	1284

**Table 2:** Statistics of annotation results.

Database	Nr	Swiss-Prot	Pfam	COG	GO	KEGG	Total
Unigenes	22,472	15,765	15,617	3261	13,702	11,970	24,317

**Table 3:** Species with matches to the annotated unigenes of *Henosepilachna vigintioctopunctata.*

Species	Unigenes Number	Percentage (%)
*Tribolium castaneum*	5069	27.73
*Anoplophora glabripennis*	3490	15.65
*Aethina tumida*	1655	7.42
*Leptinotarsa decemlineata*	1455	6.52
*Onthophagus taurus*	535	2.40
*Cryptotermes secundus*	452	2.03
*Dendroctonus ponderosae*	446	2.00
*Nicrophorus vespilloides*	364	1.63
*Agrilus planipennis*	358	1.61
*Branchiostoma belcheri*	303	1.36
*Amyelois transitella*	274	1.23
*Lasius niger*	255	1.14
*Centruroides sculpturatus*	185	0.83
*Spodoptera litura*	178	0.80
others	7285	32.66

**Table 4:** Statistics of SSRs.

SSR Information	Number
Total number of identified SSRs	4964
Number of unigenes containing SSRs	4213
Number of unigenes containing more than 1 SSR	303
Mono-nucleotide repeats	2423
Di-nucleotide repeats	1874
Tri-nucleotide repeats	631
Tetra-nucleotide repeats	30
Penta-nucleotide repeats	5
Hexa-nucleotide repeats	1

**Table 5:** Analysis software and parameters.

Database	Software	Format	Parameter
NR	DIAMOND	v0.8.37.99	1e^−5^
Swiss-Prot	DIAMOND	v0.8.37.99	1e^−5^
Pfam	HMMER3	3.1b2	default parameters
COG	DIAMOND	v0.8.37.99	1e^−5^
GO	BLAST2GO	2.5.0	default parameters
KEGG	KOBAS	2.1.1	default parameters

## Supplementary Materials

Supplementary materials can be found at https://www.mdpi.com/1422-0067/20/17/4084/s1. Figure S1. The female reproductive system of *Henosepilachna vigintioctopunctata*. A: A representative image showing the dissection of the reproductive system from a mated female in the dsGFP control group. B: A representative image showing the dissection of the reproductive system from an unmated female in the dsGFP control group. C: A representative image showing the dissection of the reproductive systems from mated females in the dsGFP, dsRC2-like, and dsPSHS-like treatment groups that did not lay eggs. Lo: lateral oviduct; Sg: spermathecal gland; Tf: terminal filament; Mo: median oviduct; Sn: seminal node; Tr: trachea; Ov: ovariole. Table S1. Gonadal development-related genes in *Henosepilachna vigintioctopunctata* ovary and testis transcriptomes. Table S2. The C_t_ values of 29 unigenes selected for RT-qPCR analysis. Table S3. Primers used in semi-RT-qPCR and RT-qPCR for validation of differentially expressed genes. Table S4. Primers used for dsRNA synthesis.

## Abbreviations

Nr	Non-redundant
COG	Clusters of Orthologous Groups of proteins
GO	Gene Ontology
KEGG	Kyoto Encyclopedia of Genes and Genomes
RT-qPCR	reverse transcriptase-quantitative polymerase chain reaction
SSRs	simple sequence repeats
