# Supplementary material for: De Novo Transcriptome Analysis Reveals Abundant Gonad-specific Genes in the Ovary and Testis of Henosepilachna vigintioctopunctata
Source: Int J Mol Sci. 2019 Aug 21;20(17):4084. doi: 10.3390/ijms20174084 (PMC6747241; doi:10.3390/ijms20174084)
Supplement: Supplementary file 1 [file ijms-20-04084-s001.pdf]

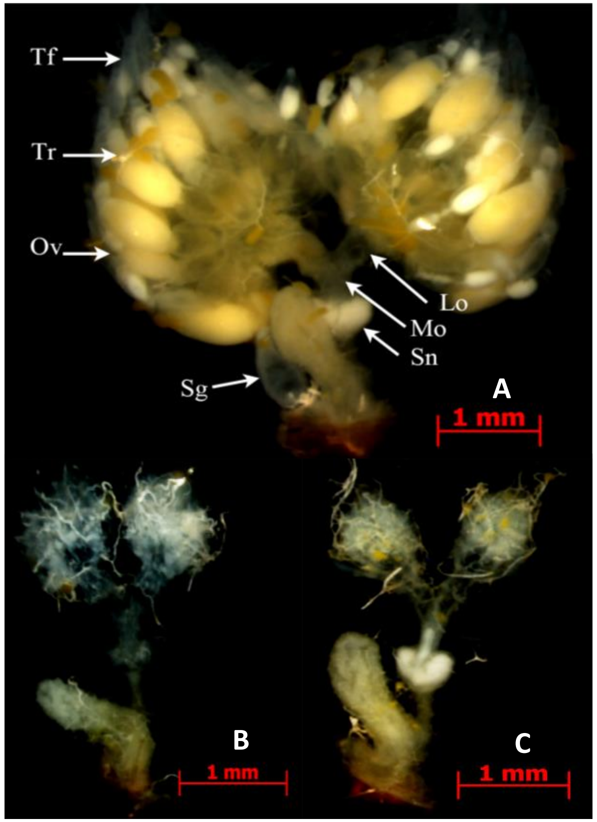

Figure S1. The female reproductive system of *H. vigintioctopunctata*. A: representative picture which the reproductive system was dissected from the mated female in the dsGFP control group. B: representative picture which the reproductive system was dissected from the unmated female in the dsGFP control group. C: representative picture which the reproductive system was dissected from the mated female in the dsGFP/ dsRC2-like/dsPSHS-like treated groups which did not lay eggs. Lo: lateral oviduct; Sg: spermathecal gland; Tf: terminal filament; Mo: median oviduct; Sn: seminal node; Tr: trachea; Ov: ovariole.

Table S1. Gonadal development related genes in *H. vigintioctopunctata* ovary and testis transcriptomes

Genes	Matched species	Length (bp)	*E* value	Identity (%)
**Oocyte meiosis**				
adenylate cyclase type 8	*Anoplophora glabripennis*	1924	2.00E-164	69.83
PREDICTED: adenylate cyclase type 2 isoform X4	*Dendroctonus ponderosae*	2992	0	70.15
PREDICTED: adenylate cyclase type 3	*Tribolium castaneum*	2838	2.00E-174	71.98
anaphase-promoting complex subunit 4	*Anoplophora glabripennis*	2666	5.00E-53	65.92
PREDICTED: anaphase-promoting complex subunit 2	*Tribolium castaneum*	3131	2.00E-169	68.43
calcium-binding protein E63-1	*Anoplophora glabripennis*	615	8.00E-30	68.73
cell division cycle protein 23 homolog	*Anoplophora glabripennis*	1849	0	73.85
cell division cycle protein 27 homolog	*Anoplophora glabripennis*	3090	5.00E-91	69.22
cell division cycle protein 16 homolog	*Anoplophora glabripennis*	1855	5.00E-139	75.56
serine/threonine-protein phosphatase 2A catalytic subunit beta isoform	*Leptinotarsa decemlineata*	2785	0	76.76
serine/threonine-protein kinase 10 isoform X3	*Anoplophora glabripennis*	4892	0	69.48
PREDICTED: serine/threonine-protein kinase mos	*Tribolium castaneum*	1708	7.00E-13	66.56

PREDICTED: serine/threonine-protein phosphatase 2A 56 kDa regulatory subunit gamma isoform isoform X4	*Tribolium castaneum*	3501	0	73.59
cAMP-dependent protein kinase catalytic subunit alpha-like	*Leptinotarsa decemlineata*	770	3.00E-28	67.8
PREDICTED: cAMP-dependent protein kinase catalytic subunit-like	*Agrilus planipennis*	1362	6.00E-103	70.96
14-3-3 protein epsilon	*Zootermopsis nevadensis*	1038	7.00E-146	75.53
14-3-3 protein zeta isoform X1	*Anoplophora glabripennis*	2161	0	83.52
structural maintenance of chromosomes protein 3	*Anoplophora glabripennis*	3851	0	75.59
PREDICTED: structural maintenance of chromosomes protein 1A	*Tribolium castaneum*	4067	0	74.69
beta-TrCP isoform X3	*Anoplophora glabripennis*	2114	0	76.69
FK506-binding protein 59	*Anoplophora glabripennis*	1584	2.00E-86	65.96
dual specificity mitogen-activated protein kinase kinase dSOR1	*Anoplophora glabripennis*	2118	0	78.13
ribosomal protein S6 kinase 2 beta isoform X2	*Anoplophora glabripennis*	3142	0	71.32
mitotic checkpoint serine/threonine-protein kinase BUB1 beta	*Anoplophora glabripennis*	3658	2.00E-98	73.21

mitogen-activated protein kinase 1	*Anoplophora glabripennis*	2301	0	77.13
separin	*Leptinotarsa decemlineata*	6148	3.00E-33	67.41
anaphase-promoting complex subunit 10	*Leptinotarsa decemlineata*	1072	2.00E-137	80.87
serine protease HTRA2, mitochondrial-like	*Leptinotarsa decemlineata*	1496	3.00E-31	72.62
rutabaga	*Tribolium castaneum*	7751	0	73.02
poly	*Onthophagus taurus*	4279	0	79.64
RING-box protein 1A	*Onthophagus taurus*	588	6.00E-89	81.16
Calmodulin	*Stylophora pistillata*	879	6.00E-05	80.77
protein phosphatase 1 catalytic subunit, partial	*Colaphellus bowringi*	1795	0	78.43
acid phosphatase, partial	*Cryptolaemus montrouzieri*	2868	0	87.05
PREDICTED: membrane-associated tyrosine- and threonine-specific cdc2-inhibitory kinase-like	*Dendroctonus ponderosae*	2365	0.004	66.06
PREDICTED: 14-3-3 protein zeta isoform X3	*Dendroctonus ponderosae*	1175	2.00E-68	80.07
PREDICTED: cell division cycle protein 16 homolog	*Aethina tumida*	689	2.00E-37	71.72
PREDICTED: cyclin-dependent kinase 2-like	*Aethina tumida*	1454	2.00E-141	72.06
PREDICTED: uncharacterized protein LOC108739266	*Agrilus planipennis*	1081	1.00E-53	74.77

PREDICTED: S-phase kinase-associated protein 1	*Agrilus planipennis*	305	6.00E-51	78.81
PREDICTED: S-phase kinase-associated protein 1 isoform X1	*Ceratosolen solmsi marchali*	845	3.00E-27	66.59
PREDICTED: inositol 1,4,5-trisphosphate receptor isoform X3	*Tribolium castaneum*	8858	0	73.04
PREDICTED: cullin-1	*Tribolium castaneum*	3231	0	74.24
PREDICTED: calcium/calmodulin-dependent protein kinase type II alpha chain isoform X10	*Tribolium castaneum*	1504	0	75.42
PREDICTED: cytoplasmic polyadenylation element-binding protein 1-B	*Tribolium castaneum*	6685	2.00E-165	76.25
**Ovarian steroidogenesis**				
insulin-like receptor	*Anoplophora glabripennis*	7550	9.00E-143	72.23
adenylate cyclase type 8	*Anoplophora glabripennis*	1924	2.00E-164	69.83
PREDICTED: adenylate cyclase type 3	*Tribolium castaneum*	2838	2.00E-174	71.98
cAMP-dependent protein kinase catalytic subunit alpha-like	*Leptinotarsa decemlineata*	770	3.00E-28	67.8
PREDICTED: cAMP-dependent protein kinase catalytic subunit-like	*Agrilus planipennis*	1362	4.00E-103	70.96

rutabaga	*Tribolium castaneum*	7751	0	73.02
PREDICTED: adenylate cyclase type 2 isoform X4	*Dendroctonus ponderosae*	2992	0	70.15
PREDICTED: guanine nucleotide-binding protein G	*Anoplophora glabripennis*	3377	1.00E-82	70.47
PREDICTED: scavenger receptor class B member 1-like	*Agrilus planipennis*	1994	6.00E-78	66.16
PREDICTED: probable cytochrome P450 303a1	*Tribolium castaneum*	795	2.00E-29	66.06
**Progesterone-mediated oocyte maturation**				
anaphase-promoting complex subunit 4	*Anoplophora glabripennis*	2666	5.00E-53	65.92
anaphase-promoting complex subunit 10	*Leptinotarsa decemlineata*	1072	2.00E-137	80.87
cell division cycle protein 23 homolog	*Anoplophora glabripennis*	1849	0	73.85
cell division cycle protein 27 homolog	*Anoplophora glabripennis*	3090	5.00E-91	69.22
heat shock protein 83-like	*Centruroides sculpturatus*	969	2.00E-135	72.55
heat shock protein 90	*Tribolium castaneum*	2435	0	76.23
PREDICTED: heat shock protein HSP 90-alpha-like	*Hyalella azteca*	1326	0	76.43
adenylate cyclase type 8	*Anoplophora glabripennis*	1924	2.00E-164	69.83
PREDICTED: adenylate cyclase type 2 isoform X4	*Dendroctonus ponderosae*	2992	0	70.15
PREDICTED: adenylate cyclase type 3	*Tribolium castaneum*	2838	2.00E-174	71.98

cAMP-dependent protein kinase catalytic subunit alpha-like	*Leptinotarsa decemlineata*	770	3.00E-28	67.8
PREDICTED: cAMP-dependent protein kinase catalytic subunit-like	*Agrilus planipennis*	1362	6.00E-103	70.96
mitogen-activated protein kinase 1	*Anoplophora glabripennis*	2301	0	77.13
mitotic checkpoint serine/threonine-protein kinase BUB1 beta	*Anoplophora glabripennis*	3658	2.00E-98	73.21
mitotic spindle assembly checkpoint protein MAD1-like	*Anoplophora glabripennis*	2604	3.00E-24	64.24
phosphatidylinositol 3-kinase regulatory subunit alpha isoform X2	*Anoplophora glabripennis*	8011	2.00E-69	64.9
probable ATP-dependent RNA helicase DDX27	*Anoplophora glabripennis*	2782	0	70.33
ribosomal protein S6 kinase 2 beta isoform X2	*Anoplophora glabripennis*	3142	0	71.32
raf homolog serine/threonine-protein kinase phl	*Anoplophora glabripennis*	3433	0	73.44
dual specificity mitogen-activated protein kinase kinase dSOR1	*Anoplophora glabripennis*	2118	0	78.13
rutabaga	*Tribolium castaneum*	7751	0	73.02
RAC serine/threonine-protein kinase isoform X2	*Leptinotarsa decemlineata*	2024	0	73.09
RNA-binding protein 41	*Harmonia axyridis*	2116	5.00E-20	65.35

protein enhancer of sevenless 2B	*Zootermopsis nevadensis*	1362	4.00E-36	67.17
PREDICTED: cell division cycle protein 16 homolog	*Aethina tumida*	689	2.00E-37	71.72
PREDICTED: cyclin-dependent kinase 2-like	*Aethina tumida*	1454	2.00E-141	72.06
PREDICTED: uncharacterized protein LOC108739266	*Agrilus planipennis*	1081	1.00E-53	74.77
PREDICTED: fizzy-related protein homolog	*Tribolium castaneum*	3134	0	73.3
PREDICTED: cytoplasmic polyadenylation element-binding protein 1-B	*Tribolium castaneum*	6685	2.00E-165	76.25
PREDICTED: anaphase-promoting complex subunit 2	*Tribolium castaneum*	3131	2.00E-169	68.43
PREDICTED: G protein alpha i subunit	*Tribolium castaneum*	3106	0	73.79
PREDICTED: serine/threonine-protein kinase mos	*Tribolium castaneum*	1708	7.00E-13	66.56
**Estrogen signaling pathway**				
testis-specific heat shock protein cognate 70-6	*Laodelphax striatella*	367	3.00E-13	73.28
heat shock protein 68	*Harmonia axyridis*	2242	0	78.25
heat shock protein 70	*Harmonia axyridis*	3004	0	73.57
heat shock protein 68-like	*Leptinotarsa decemlineata*	1720	0	75.03
heat shock protein 83-like	*Centruroides sculpturatus*	969	2.00E-135	72.55

heat shock protein 90	*Tribolium castaneum*	2435	0	76.23
heat shock 70 kDa protein cognate 4-like	*Centruroides sculpturatus*	2161	0	71.64
PREDICTED: heat shock 70 kDa protein cognate 2	*Tribolium castaneum*	744	8.00E-141	78.17
PREDICTED: heat shock 70 kDa protein cognate 4 isoform X2	*Megachile rotundata*	312	3.00E-23	85.11
PREDICTED: heat shock protein HSP 90-alpha-like	*Hyalella azteca*	1326	0	76.43
adenylate cyclase type 8	*Anoplophora glabripennis*	1924	2.00E-164	69.83
PREDICTED: adenylate cyclase type 3	*Tribolium castaneum*	2838	2.00E-174	71.98
calcium-binding protein E63-1	*Anoplophora glabripennis*	615	8.00E-30	68.73
cyclic AMP-responsive element-binding protein 3-like protein 1	*Anoplophora glabripennis*	2996	3.00E-55	72.26
dual specificity mitogen-activated protein kinase kinase dSOR1	*Anoplophora glabripennis*	2118	0	78.13
mitogen-activated protein kinase 1	*Anoplophora glabripennis*	2301	0	77.13
rutabaga	*Tribolium castaneum*	7751	0	73.02
phosphatidylinositol 3-kinase regulatory subunit alpha isoform X2	*Anoplophora glabripennis*	8011	2.00E-69	64.9

G-protein alpha subunit	*Lissorhoptrus oryzophilus*	2446	0	79.53
RAC serine/threonine-protein kinase isoform X2	*Leptinotarsa decemlineata*	2024	0	73.09
RNA-binding protein 41	*Harmonia axyridis*	2116	5.00E-20	65.35
FK506-binding protein 59	*Anoplophora glabripennis*	1584	2.00E-86	65.96
uncharacterized protein LOC111511578	*Leptinotarsa decemlineata*	988	2.00E-08	69.54
protein son of sevenless isoform X2	*Leptinotarsa decemlineata*	5093	0	71.23
cAMP-dependent protein kinase catalytic subunit alpha-like	*Leptinotarsa decemlineata*	770	3.00E-28	67.8
Calmodulin	*Stylophora pistillata*	879	6.00E-05	80.77
PREDICTED: cAMP-dependent protein kinase catalytic subunit-like	*Agrilus planipennis*	1362	6.00E-103	70.96
PREDICTED: LOW QUALITY PROTEIN: heat shock protein 68-like	*Aethina tumida*	1043	7.00E-146	73.66
PREDICTED: protein enhancer of sevenless 2B	*Tribolium castaneum*	636	4.00E-55	82.55
PREDICTED: inositol 1,4,5-trisphosphate receptor isoform X3	*Tribolium castaneum*	8858	0	73.04
PREDICTED: G protein alpha i subunit	*Tribolium castaneum*	3106	0	73.79

PREDICTED: SHC-transforming protein 1	*Tribolium castaneum*	5140	4.00E-130	69.36
PREDICTED: guanine nucleotide-binding protein G	*Tribolium castaneum*	4488	0	79.31
PREDICTED: endoplasmin	*Linepithema humile*	2719	6.00E-174	67.15
PREDICTED: adenylate cyclase type 2 isoform X4	*Dendroctonus ponderosae*	2992	0	70.15
**Prolactin signaling pathway**				
dual specificity mitogen-activated protein kinase kinase dSOR1	*Anoplophora glabripennis*	2118	0	78.13
mitogen-activated protein kinase 1	*Anoplophora glabripennis*	2301	0	77.13
phosphatidylinositol 3-kinase regulatory subunit alpha isoform X2	*Anoplophora glabripennis*	8011	2.00E-69	64.9
protein son of sevenless isoform X2	*Leptinotarsa decemlineata*	5093	0	71.23
RAC serine/threonine-protein kinase isoform X2	*Leptinotarsa decemlineata*	2024	0	73.09
tyrosine hydroxylase	*Tribolium castaneum*	2123	0	69.58
RNA-binding protein 41	*Harmonia axyridis*	2116	5.00E-20	65.35
suppressor of cytokine signaling 2	*Harmonia axyridis*	2643	2.00E-114	70.63
PREDICTED: G1/S-specific cyclin-D3-like	*Aethina tumida*	1476	3.00E-44	68
PREDICTED: nuclear factor NF-kappa-B p110 subunit	*Tribolium castaneum*	3301	3.00E-78	68.03

isoform X1				
PREDICTED: SHC-transforming protein 1	*Tribolium castaneum*	5140	4.00E-130	69.36
PREDICTED: uncharacterized protein LOC662537	*Tribolium castaneum*	2939	5.00E-114	77.86
PREDICTED: BAG family molecular chaperone regulator 2	*Tribolium castaneum*	851	3.00E-71	71.92
PREDICTED: protein enhancer of sevenless 2B	*Tribolium castaneum*	636	4.00E-55	82.55
**Thyroid hormone synthesis**				
adenylate cyclase type 8	*Anoplophora glabripennis*	1924	2.00E-164	69.83
PREDICTED: adenylate cyclase type 2 isoform X4	*Dendroctonus ponderosae*	2992	0	70.15
iodotyrosine deiodinase 1 isoform X1	*Anoplophora glabripennis*	1025	8.00E-64	70.48
cyclic AMP-responsive element-binding protein 3-like protein 1	*Anoplophora glabripennis*	2996	3.00E-55	72.26
low-density lipoprotein receptor class A domain-containing protein 3-like	*Anoplophora glabripennis*	2634	1.00E-54	71.91
rutabaga	*Tribolium castaneum*	7751	0	73.02
Protein Wnt-2-like Protein	*Tribolium castaneum*	2691	0	73.94
transcription termination factor 2-like	*Onthophagus taurus*	3187	2.00E-27	64.16

cAMP-dependent protein kinase catalytic subunit alpha-like	*Leptinotarsa decemlineata*	770	3.00E-28	67.8
PREDICTED: cAMP-dependent protein kinase catalytic subunit-like	*Agrilus planipennis*	1362	6.00E-103	70.96
PREDICTED: endoplasmin	*Linepithema humile*	2719	6.00E-174	67.15
PREDICTED: LOW QUALITY PROTEIN: sodium/potassium-transporting ATPase subunit beta-2-like	*Aethina tumida*	2892	2.00E-25	67.53
PREDICTED: probable phospholipid hydroperoxide glutathione peroxidase isoform X1	*Aethina tumida*	1230	2.00E-57	70.85
PREDICTED: adenylate cyclase type 3	*Tribolium castaneum*	2838	2.00E-174	71.98
PREDICTED: guanine nucleotide-binding protein G	*Tribolium castaneum*	4488	0	79.31
PREDICTED: inositol 1,4,5-trisphosphate receptor isoform X3	*Tribolium castaneum*	8858	0	73.04
**Thyroid hormone signaling pathway**				
testis-specific actin	*Rhipicephalus appendiculatus*	298	8.00E-85	82.83
actin, cytoplasmic A3a	*Ceratitis capitata*	1195	0	81.67
beta-actin	*Tigriopus japonicus*	1771	9.00E-173	90.07

PREDICTED: actin, cytoplasmic	*Tribolium castaneum*	667	1.00E-92	77.91
mitogen-activated protein kinase 1	*Anoplophora glabripennis*	2301	0	77.13
mediator of RNA polymerase II transcription subunit 13 isoform X3	*Anoplophora glabripennis*	7898	0	70.55
PREDICTED: mediator of RNA polymerase II transcription subunit 16 isoform X1	*Tribolium castaneum*	3031	0	67.7
PREDICTED: mediator of RNA polymerase II transcription subunit 17 isoform X2	*Tribolium castaneum*	2263	0	74.06
PREDICTED: mediator of RNA polymerase II transcription subunit 24	*Tribolium castaneum*	3506	0	68.31
solute carrier family 2, facilitated glucose transporter member 1	*Anoplophora glabripennis*	2568	2.00E-82	65.71
PREDICTED: solute carrier family 2, facilitated glucose transporter member 3-like isoform X2	*Ceratosolen solmsi marchali*	845	3.00E-14	67.93
histone deacetylase Rpd3 isoform X1	*Anoplophora glabripennis*	1908	0	78.01
hypoxia-inducible factor 1-alpha isoform X2	*Anoplophora glabripennis*	2366	2.00E-146	70.95
phosphatidylinositol 3-kinase regulatory subunit alpha isoform X2	*Anoplophora glabripennis*	8011	2.00E-69	64.9

protein decapentaplegic	*Anoplophora glabripennis*	3840	8.00E-52	74.03
dual specificity mitogen-activated protein kinase kinase dSOR1	*Anoplophora glabripennis*	2118	0	78.13
transcriptional regulator Myc-1 isoform X2	*Anoplophora glabripennis*	3653	1.00E-24	74.18
6-phosphofructo-2-kinase/fructose-2,6-bisphosphatase 1 isoform X1	*Anoplophora glabripennis*	3057	0	72.49
RNA-binding protein 41	*Harmonia axyridis*	2116	5.00E-20	65.35
ultraspiracle 2 isoform	*Henosepilachna vigintioctopunctata*	2221	0	99.56
uncharacterized protein LOC111510921	*Leptinotarsa decemlineata*	1310	1.00E-17	74.34
mediator of RNA polymerase II transcription subunit 30-like	*Leptinotarsa decemlineata*	1185	1.00E-97	76.1
RAC serine/threonine-protein kinase isoform X2	*Leptinotarsa decemlineata*	2024	0	73.09
glucose transporter type 1 isoform X4	*Onthophagus taurus*	2680	0	74.83
ras-like GTP-binding protein RHO	*Orbicella faveolata*	854	2.00E-10	83.08
steroid receptor coactivator	*Monochamus alternatus*	6138	0	67.81
cAMP-dependent protein kinase catalytic subunit alpha-like	*Leptinotarsa decemlineata*	770	3.00E-28	67.8

PREDICTED: cAMP-dependent protein kinase catalytic subunit-like	*Agrilus planipennis*	1362	6.00E-103	70.96
PREDICTED: LOW QUALITY PROTEIN: histone deacetylase 3-like	*Aethina tumida*	3494	0	77.72
PREDICTED: LOW QUALITY PROTEIN: sodium/potassium-transporting ATPase subunit beta-2-like	*Aethina tumida*	2892	2.00E-25	67.53
PREDICTED: 1-phosphatidylinositol 4,5-bisphosphate phosphodiesterase gamma-1	*Tribolium castaneum*	4244	0	69.03
PREDICTED: 3-phosphoinositide-dependent protein kinase 1	*Tribolium castaneum*	1906	6.00E-27	67.01
PREDICTED: GATA-binding factor A isoform X2	*Tribolium castaneum*	2797	3.00E-109	72.24
PREDICTED: transcription factor GATA-4 isoform X1	*Tribolium castaneum*	2657	4.00E-88	80.34
PREDICTED: GTP-binding protein Rheb homolog	*Nicrophorus vespilloides*	2052	3.00E-97	76.4
**Oxytocin signaling pathway**				
testis-specific actin	*Rhipicephalus appendiculatus*	298	8.00E-85	82.83
actin, cytoplasmic A3a	*Ceratitis capitata*	1195	0	81.67
beta-actin	*Tigriopus japonicus*	1771	9.00E-173	90.07

PREDICTED: actin, cytoplasmic	*Tribolium castaneum*	667	1.00E-92	77.91
PREDICTED: testis-specific muscle calcium channel subunit alpha-1 isoform X2	*Nicrophorus vespilloides*	439	8.00E-53	75.83
protein phosphatase 1 catalytic subunit, partial	*Colaphellus bowringi*	1795	0	78.43
PREDICTED: protein phosphatase 1 regulatory subunit 12B isoform X4	*Tribolium castaneum*	3143	0	69.69
adenylate cyclase type 8	*Anoplophora glabripennis*	1924	2.00E-164	69.83
PREDICTED: adenylate cyclase type 2 isoform X4	*Dendroctonus ponderosae*	2992	0	70.15
PREDICTED: adenylate cyclase type 3	*Tribolium castaneum*	2838	2.00E-174	71.98
calcium-binding protein E63-1	*Anoplophora glabripennis*	615	8.00E-30	68.73
PREDICTED: calcium/calmodulin-dependent protein kinase type 1	*Tribolium castaneum*	3330	0	79.48
PREDICTED: calcium/calmodulin-dependent protein kinase type II alpha chain isoform X10	*Tribolium castaneum*	1504	0	75.42
cAMP-dependent protein kinase catalytic subunit alpha-like	*Leptinotarsa decemlineata*	770	3.00E-28	67.8
PREDICTED: cAMP-dependent protein kinase catalytic subunit-like	*Agrilus planipennis*	1362	6.00E-103	70.96

G-protein alpha subunit	*Lissorhoptrus oryzophilus*	2446	0	79.53
PREDICTED: G protein alpha i subunit	*Tribolium castaneum*	3106	0	73.79
FK506-binding protein 59	*Anoplophora glabripennis*	1584	2.00E-86	65.96
titin	*Anoplophora glabripennis*	3672	0	71.6
mitogen-activated protein kinase 1	*Anoplophora glabripennis*	2301	0	77.13
dual specificity mitogen-activated protein kinase kinase dSOR1	*Anoplophora glabripennis*	2118	0	78.13
5'-AMP-activated protein kinase catalytic subunit alpha-2	*Dastarcus helophoroides*	2267	0	77.07
hypothetical protein YQE_10269, partial	*Dendroctonus ponderosae*	3681	1.00E-118	71.4
rutabaga	*Tribolium castaneum*	7751	0	73.02
ryanodine receptor	*Tribolium castaneum*	10129	0	74.66
acid phosphatase, partial	*Cryptolaemus montrouzieri*	2868	0	87.05
Calmodulin	*Stylophora pistillata*	879	6.00E-05	80.77
PREDICTED: guanine nucleotide-binding protein G	*Tribolium castaneum*	4488	0	79.31
PREDICTED: inositol 1,4,5-trisphosphate receptor isoform X3	*Tribolium castaneum*	8858	0	73.04

PREDICTED: head-specific guanylate cyclase	*Tribolium castaneum*	1161	2.00E-175	76.11
**GnRH signaling pathway**				
testis-specific PREDICTED: muscle calcium channel subunit alpha-1 isoform X2	*Nicrophorus vespilloides*	439	8.00E-53	75.83
testis-specific PREDICTED: cell division control protein 42 homolog	*Branchiostoma belcheri*	310	1.00E-30	80.42
adenylate cyclase type 8	*Anoplophora glabripennis*	1924	2.00E-164	69.83
PREDICTED: adenylate cyclase type 2 isoform X4	*Dendroctonus ponderosae*	2992	0	70.15
PREDICTED: adenylate cyclase type 3	*Tribolium castaneum*	2838	2.00E-174	71.98
cAMP-dependent protein kinase catalytic subunit alpha-like	*Leptinotarsa decemlineata*	770	3.00E-28	67.8
PREDICTED: cAMP-dependent protein kinase catalytic subunit-like	*Agrilus planipennis*	1362	6.00E-103	70.96
matrix metalloproteinase 1 isoform 2 precursor	*Tribolium castaneum*	2544	7.00E-143	68.48
PREDICTED: matrix metalloproteinase-14-like isoform X1	*Nicrophorus vespilloides*	1143	1.00E-24	71.07
protein enhancer of sevenless 2B	*Zootermopsis nevadensis*	1362	4.00E-36	67.17
PREDICTED: protein enhancer of sevenless 2B	*Tribolium castaneum*	636	4.00E-55	82.55

calcium-binding protein E63-1	*Anoplophora glabripennis*	615	8.00E-30	68.73
dual specificity mitogen-activated protein kinase kinase dSOR1	*Anoplophora glabripennis*	2118	0	78.13
mitogen-activated protein kinase 1	*Anoplophora glabripennis*	2301	0	77.13
rutabaga	*Tribolium castaneum*	7751	0	73.02
DNA excision repair protein ERCC-6-like isoform X3	*Leptinotarsa decemlineata*	1619	1.00E-115	70.59
protein son of sevenless isoform X2	*Leptinotarsa decemlineata*	5093	0	71.23
Calmodulin	*Stylophora pistillata*	879	6.00E-05	80.77
PREDICTED: calcium/calmodulin-dependent protein kinase type II alpha chain isoform X10	*Tribolium castaneum*	1504	0	75.42
PREDICTED: guanine nucleotide-binding protein G	*Tribolium castaneum*	4488	0	79.31
PREDICTED: inositol 1,4,5-trisphosphate receptor isoform X3	*Tribolium castaneum*	8858	0	73.04
PREDICTED: cdc42 homolog	*Tribolium castaneum*	1786	3.00E-138	78.99
**Apoptosis**				
testis-specific probable multidrug resistance-associated protein lethal	*Diabrotica virgifera virgifera*	929	6.00E-39	73.18

testis-specific Cathepsin L1 precursor	*Caligus clemensi*	385	1.00E-31	73.61
testis-specific PREDICTED: cathepsin L1-like	*Aethina tumida*	1122	5.00E-15	67.32
testis-specific actin	*Rhipicephalus appendiculatus*	298	8.00E-85	82.83
actin, cytoplasmic A3a	*Ceratitis capitata*	1195	0	81.67
beta-actin	*Tigriopus japonicus*	1771	9.00E-173	90.07
PREDICTED: actin, cytoplasmic	*Tribolium castaneum*	667	1.00E-92	77.91
dual specificity mitogen-activated protein kinase kinase dSOR1	*Anoplophora glabripennis*	2118	0	78.13
mitogen-activated protein kinase 1	*Anoplophora glabripennis*	2301	0	77.13
tubulin alpha-4 chain-like	*Anoplophora glabripennis*	1526	0	74.4
phosphatidylinositol 3-kinase regulatory subunit alpha isoform X2	*Anoplophora glabripennis*	8011	2.00E-69	64.9
RNA-binding protein 41	*Harmonia axyridis*	2116	5.00E-20	65.35
RAC serine/threonine-protein kinase isoform X2	*Leptinotarsa decemlineata*	2024	0	73.09
growth arrest and DNA damage-inducible protein GADD45 alpha	*Leptinotarsa decemlineata*	1125	2.00E-31	70.63
serine protease HTRA2, mitochondrial-like	*Leptinotarsa decemlineata*	1496	3.00E-31	72.62

PREDICTED: 3-phosphoinositide-dependent protein kinase 1	*Tribolium castaneum*	1906	6.00E-27	67.01
PREDICTED: nuclear factor NF-kappa-B p110 subunit isoform X1	*Tribolium castaneum*	3301	3.00E-78	68.03
PREDICTED: inositol 1,4,5-trisphosphate receptor isoform X3	*Tribolium castaneum*	8858	0	73.04
**Apoptosis - fly**				
raf homolog serine/threonine-protein kinase phl	*Anoplophora glabripennis*	3433	0	73.44
ultraspiracle 2 isoform	*Henosepilachna vigintioctopunctata*	2221	0	99.56
mitogen-activated protein kinase 1	*Anoplophora glabripennis*	2301	0	77.13
PREDICTED: mitogen-activated protein kinase kinase kinase 7-like isoform X1	*Agrilus planipennis*	1968	1.00E-74	69.19
serine protease HTRA2, mitochondrial-like	*Leptinotarsa decemlineata*	1496	3.00E-31	72.62
PREDICTED: serine/threonine-protein kinase 3 isoform X1	*Tribolium castaneum*	4285	0	73.78
**Apoptosis - multiple species**				
PREDICTED: S-phase kinase-associated protein 1 isoform X1	*Ceratosolen solmsi marchali*	845	3.00E-27	66.59

Steroid hormone biosynthesis

testis-specific UDP-glucuronosyltransferase 2B16-like isoform X1	*Anoplophora glabripennis*	629	3.00E-35	69.8
testis-specific PREDICTED: UDP-glucuronosyltransferase 2B2-like	*Nicrophorus vespilloides*	477	1.00E-06	80
uncharacterized protein LOC108903775	*Anoplophora glabripennis*	1273	9.00E-14	67.77
uncharacterized protein LOC108904558	*Anoplophora glabripennis*	1755	4.00E-07	77.11
uncharacterized protein LOC111515682	*Leptinotarsa decemlineata*	1338	2.00E-45	70.79
hypothetical protein D910_12170	*Dendroctonus ponderosae*	1589	7.00E-50	69.23
hypothetical protein TcasGA2_TC012282	*Tribolium castaneum*	1657	5.00E-14	65.04
UDP-glucuronosyltransferase 2B19 isoform X1	*Anoplophora glabripennis*	1995	5.00E-64	65.95
UDP-glucuronosyltransferase 2A3-like isoform X1	*Leptinotarsa decemlineata*	1930	1.00E-11	70.99
very-long-chain 3-oxoacyl-CoA reductase-B	*Leptinotarsa decemlineata*	1283	1.00E-48	65.71
PREDICTED: UDP-glucuronosyltransferase 1-1-like	*Agrilus planipennis*	660	1.00E-05	81.13
PREDICTED: UDP-glucuronosyltransferase 1-7 isoform X3	*Tribolium castaneum*	1323	4.00E-40	72.39
PREDICTED: UDP-glucuronosyltransferase 1-7C	*Tribolium castaneum*	834	8.00E-16	70.21

isoform X4				
PREDICTED: UDP-glucuronosyltransferase 2B7-like	*Aethina tumida*	1878	3.00E-21	67.82
PREDICTED: UDP-glucuronosyltransferase 2B7 isoform X2	*Tribolium castaneum*	1670	6.00E-13	68.69
PREDICTED: UDP-glucuronosyltransferase 2B9	*Tribolium castaneum*	1250	2.00E-05	81.82
PREDICTED: UDP-glucuronosyltransferase 2B14	*Tribolium castaneum*	1913	8.00E-19	76.34
PREDICTED: UDP-glucuronosyltransferase 2B15-like	*Agrilus planipennis*	417	1.00E-11	76.29
PREDICTED: UDP-glucuronosyltransferase 2B17-like	*Dendroctonus ponderosae*	483	4.00E-07	71.3
PREDICTED: UDP-glucuronosyltransferase 2C1-like	*Tribolium castaneum*	1523	7.00E-26	72.35
PREDICTED: 2-hydroxyacylsphingosine 1-beta-galactosyltransferase-like	*Tribolium castaneum*	1795	1.00E-29	69.45
**Homologous recombination**				
testis-specific PREDICTED: uncharacterized protein LOC106134883	*Amyelois transitella*	414	1.00E-135	85.82
testis-specific PREDICTED: uncharacterized protein LOC106140328	*Amyelois transitella*	271	6.00E-73	82.72
testis-specific PREDICTED: uncharacterized protein LOC109038256	*Bemisia tabaci*	336	6.00E-10	71.9

DNA repair protein RAD50 isoform X1	*Anoplophora glabripennis*	4231	7.00E-34	68.14
DNA repair protein RAD51 homolog 1	*Leptinotarsa decemlineata*	1294	0	76.06
DNA repair and recombination protein RAD54-like	*Anoplophora glabripennis*	429	6.00E-29	72.73
DNA topoisomerase 3-beta-1	*Anoplophora glabripennis*	3145	0	73.66
DNA polymerase delta small subunit	*Anoplophora glabripennis*	1805	2.00E-74	66.88
BRISC and BRCA1-A complex member 2	*Anoplophora glabripennis*	2183	8.00E-24	66.28
breast cancer type 2 susceptibility protein-like isoform X2	*Anoplophora glabripennis*	4335	1.00E-24	67.31
uncharacterized protein LOC111615589	*Centruroides sculpturatus*	1093	1.00E-05	68.66
PREDICTED: uncharacterized protein LOC106131883 isoform X1	*Amyelois transitella*	470	4.00E-28	69.23
PREDICTED: crossover junction endonuclease EME1	*Agrilus planipennis*	1985	2.00E-16	66.83
PREDICTED: single-stranded DNA-binding protein, mitochondrial	*Tribolium castaneum*	1323	4.00E-59	76.23
**MAPK signaling pathway**				
testis-specific mitogen-activated protein kinase kinase kinase 13-A	*Leptinotarsa decemlineata*	817	5.00E-07	72.73

testis-specific heat shock protein cognate 70-6	*Laodelphax striatella*	367	3.00E-13	73.28
testis-specific PREDICTED: cell division control protein 42 homolog	*Branchiostoma belcheri*	310	1.00E-30	80.42
testis-specific PREDICTED: TGF-beta receptor type-1 isoform X3	*Nicrophorus vespilloides*	353	2.00E-08	72.13
testis-specific PREDICTED: muscle calcium channel subunit alpha-1 isoform X2	*Nicrophorus vespilloides*	439	8.00E-53	75.83
heat shock protein 68	*Harmonia axyridis*	2242	0	78.25
heat shock protein 70	*Harmonia axyridis*	3004	0	73.57
heat shock protein 68-like	*Leptinotarsa decemlineata*	1720	0	75.03
PREDICTED: heat shock 70 kDa protein cognate 2	*Tribolium castaneum*	744	8.00E-141	78.17
PREDICTED: heat shock 70 kDa protein cognate 4 isoform X2	*Megachile rotundata*	312	3.00E-23	85.11
cAMP-dependent protein kinase catalytic subunit alpha-like	*Leptinotarsa decemlineata*	770	1.00E-28	67.8
PREDICTED: cAMP-dependent protein kinase catalytic subunit-like	*Agrilus planipennis*	1362	6.00E-103	70.96
mitogen-activated protein kinase 1	*Anoplophora glabripennis*	2301	0	77.13

PREDICTED: mitogen-activated protein kinase kinase kinase 7-like isoform X1	*Agrilus planipennis*	1968	1.00E-74	69.19
dual specificity mitogen-activated protein kinase kinase dSOR1	*Anoplophora glabripennis*	2118	0	78.13
PREDICTED: dual specificity protein phosphatase 3	*Tribolium castaneum*	914	8.00E-53	68.37
MAP kinase-activated protein kinase 2	*Anoplophora glabripennis*	2384	0	77.83
MAP kinase-interacting serine/threonine-protein kinase 1	*Anoplophora glabripennis*	880	1.00E-117	76.35
TGF-beta-activated kinase 1 and MAP3K7-binding protein 1	*Anoplophora glabripennis*	3006	8.00E-57	69.07
transcriptional regulator Myc-1 isoform X2	*Anoplophora glabripennis*	3653	1.00E-24	74.18
ras-related C3 botulinum toxin substrate 1	*Anoplophora glabripennis*	2805	4.00E-136	78.21
raf homolog serine/threonine-protein kinase phl	*Anoplophora glabripennis*	3433	0	73.44
ribosomal protein S6 kinase 2 beta isoform X2	*Anoplophora glabripennis*	3142	0	71.32
DNA excision repair protein ERCC-6-like isoform X3	*Leptinotarsa decemlineata*	1619	1.00E-115	70.59
growth arrest and DNA damage-inducible protein GADD45 alpha	*Leptinotarsa decemlineata*	1125	2.00E-31	70.63
RAC serine/threonine-protein kinase isoform X2	*Leptinotarsa decemlineata*	2024	0	73.09

protein son of sevenless isoform X2	*Leptinotarsa decemlineata*	5093	0	71.23
protein enhancer of sevenless 2B	*Zootermopsis nevadensis*	1362	4.00E-36	67.17
RNA-binding protein 41	*Harmonia axyridis*	2116	5.00E-20	65.35
PREDICTED: LOW QUALITY PROTEIN: ras GTPase-activating protein 1-like	*Aethina tumida*	4518	0	74.04
PREDICTED: LOW QUALITY PROTEIN: heat shock protein 68-like	*Aethina tumida*	1043	7.00E-146	73.66
PREDICTED: zinc finger protein 2 homolog isoform X1	*Tribolium castaneum*	4028	2.00E-67	74.49
PREDICTED: serine/threonine-protein kinase mos	*Tribolium castaneum*	1708	7.00E-13	66.56
PREDICTED: serine/threonine-protein kinase 3 isoform X1	*Tribolium castaneum*	4285	0	73.78
PREDICTED: serine/threonine-protein kinase NLK isoform X1	*Tribolium castaneum*	3626	0	78.33
PREDICTED: serine/threonine-protein phosphatase 5	*Tribolium castaneum*	1774	0	71.26
PREDICTED: protein max isoform X3	*Tribolium castaneum*	1363	5.00E-70	73.66
PREDICTED: protein enhancer of sevenless 2B	*Tribolium castaneum*	636	4.00E-55	82.55
PREDICTED: adapter molecule Crk	*Tribolium castaneum*	1329	2.00E-128	72.72

PREDICTED: ras-like protein 3	*Tribolium castaneum*	4357	0	78.58
PREDICTED: nuclear factor NF-kappa-B p110 subunit isoform X1	*Tribolium castaneum*	3301	3.00E-78	68.03
PREDICTED: cdc42 homolog	*Tribolium castaneum*	1786	3.00E-138	78.99
PREDICTED: protein phosphatase 1A	*Tribolium castaneum*	4182	3.00E-168	74.23
PREDICTED: fizzy-related protein homolog	*Tribolium castaneum*	3134	0	73.3
**MAPK signaling pathway - fly**				
testis-specific PREDICTED: leucine-rich repeat-containing protein 24-like	*Nicrophorus vespilloides*	519	2.00E-11	72.44
sprouty	*Harmonia axyridis*	2358	0	82.6
Agrilus planipennis	*Agrilus planipennis*	2005	0	79.81
protein trunk	*Anoplophora glabripennis*	1337	2.00E-04	67.72
protein decapentaplegic	*Anoplophora glabripennis*	3840	8.00E-52	74.03
protein C-ets-2 isoform X1	*Anoplophora glabripennis*	3507	0	70.56
protein enhancer of sevenless 2B	*Zootermopsis nevadensis*	1362	4.00E-36	67.17
protein son of sevenless isoform X2	*Leptinotarsa decemlineata*	5093	0	71.23
raf homolog serine/threonine-protein kinase phl	*Anoplophora glabripennis*	3433	0	73.44

ras-related C3 botulinum toxin substrate 1	*Anoplophora glabripennis*	2805	4.00E-136	78.21
mitogen-activated protein kinase 1	*Anoplophora glabripennis*	2301	0	77.13
PREDICTED: mitogen-activated protein kinase kinase kinase 7-like isoform X1	*Agrilus planipennis*	1968	1.00E-74	69.19
dual specificity mitogen-activated protein kinase kinase dSOR1	*Anoplophora glabripennis*	2118	0	78.13
E3 ubiquitin-protein ligase SIAH1-like	*Anoplophora glabripennis*	1012	2.00E-15	65.37
receptor tyrosine kinase Torso-like protein	*Tribolium castaneum*	944	5.00E-05	72.63
uncharacterized protein LOC111504284	*Leptinotarsa decemlineata*	3305	4.00E-154	70.35
serine/threonine-protein phosphatase 2A catalytic subunit beta isoform	*Leptinotarsa decemlineata*	2785	0	76.76
DNA excision repair protein ERCC-6-like isoform X3	*Leptinotarsa decemlineata*	1619	1.00E-115	70.59
PREDICTED: E3 ubiquitin-protein ligase SIAH1-like	*Aethina tumida*	2663	4.00E-172	77.66
PREDICTED: LOW QUALITY PROTEIN: ras GTPase-activating protein 1-like	*Aethina tumida*	4518	0	74.04
PREDICTED: LOW QUALITY PROTEIN: exportin-1	*Aethina tumida*	4508	0	71.34
PREDICTED: 14-3-3 protein zeta isoform X3	*Dendroctonus ponderosae*	1175	2.00E-68	80.07

PREDICTED: protein phosphatase 1A	*Tribolium castaneum*	4182	3.00E-168	74.23
PREDICTED: protein groucho isoform X4	*Tribolium castaneum*	2750	0	72.27
PREDICTED: tyrosine-protein kinase Btk29A isoform X1	*Tribolium castaneum*	2685	0	76.34
PREDICTED: SHC-transforming protein 1	*Tribolium castaneum*	5140	4.00E-130	69.36
PREDICTED: modulator of activity of ets isoform X1	*Tribolium castaneum*	1018	3.00E-15	68.3
PREDICTED: homeobox protein prospero isoform X2	*Tribolium castaneum*	3643	0	72.74
PREDICTED: zinc finger protein 2 homolog isoform X1	*Tribolium castaneum*	4028	2.00E-67	74.49
**Ras signaling pathway**				
testis-specific PREDICTED: LOW QUALITY PROTEIN: protein still life, isoform SIF type 1-like	*Aethina tumida*	2389	2.00E-29	74.07
testis-specific PREDICTED: protein still life, isoform SIF type 1 isoform X13	*Tribolium castaneum*	472	5.00E-98	76.91
testis-specific PREDICTED: cell division control protein 42 homolog	*Branchiostoma belcheri*	310	1.00E-30	80.42
cAMP-dependent protein kinase catalytic subunit alpha-like	*Leptinotarsa decemlineata*	770	3.00E-28	67.8
PREDICTED: cAMP-dependent protein kinase catalytic subunit-like	*Agrilus planipennis*	1362	6.00E-103	70.96

ras-like GTP-binding protein RHO	*Orbicella faveolata*	854	2.00E-10	83.08
PREDICTED: ras-like protein 2	*Harmonia axyridis*	430	0.008	92.59
PREDICTED: ras-like protein 3	*Tribolium castaneum*	4357	0	78.58
ras-related C3 botulinum toxin substrate 1	*Anoplophora glabripennis*	2805	4.00E-136	78.21
PREDICTED: ras-related protein Ral-a	*Tribolium castaneum*	2275	3.00E-100	73.32
mitogen-activated protein kinase 1	*Anoplophora glabripennis*	2301	0	77.13
5-hydroxytryptamine receptor 1-like	*Anoplophora glabripennis*	2157	0	73.52
leucine-rich repeat protein soc-2 homolog	*Anoplophora glabripennis*	3775	0	71.46
protein C-ets-2 isoform X1	*Anoplophora glabripennis*	3507	0	70.56
dual specificity mitogen-activated protein kinase kinase dSOR1	*Anoplophora glabripennis*	2118	0	78.13
vascular endothelial growth factor receptor 1 isoform X4	*Anoplophora glabripennis*	3248	1.00E-42	73.52
phosphatidylinositol 3-kinase regulatory subunit alpha isoform X2	*Anoplophora glabripennis*	8011	2.00E-69	64.9
BRCA1-associated protein isoform X2	*Anoplophora glabripennis*	2261	2.00E-165	69.74
insulin-like receptor	*Anoplophora glabripennis*	7550	9.00E-143	72.23
calcium-binding protein E63-1	*Anoplophora glabripennis*	615	8.00E-30	68.73

RNA-binding protein 41	*Harmonia axyridis*	2116	5.00E-20	65.35
protein son of sevenless isoform X2	*Leptinotarsa decemlineata*	5093	0	71.23
RAC serine/threonine-protein kinase isoform X2	*Leptinotarsa decemlineata*	2024	0	73.09
Calmodulin	*Stylophora pistillata*	879	6.00E-05	80.77
PREDICTED: vascular endothelial growth factor receptor 1 isoform X1	*Tribolium castaneum*	1926	2.00E-21	66.57
PREDICTED: vascular endothelial growth factor receptor 1 isoform X2	*Megachile rotundata*	465	3.00E-20	71.96
PREDICTED: LOW QUALITY PROTEIN: ras GTPase-activating protein 1-like	*Aethina tumida*	4518	0	74.04
PREDICTED: protein enhancer of sevenless 2B	*Tribolium castaneum*	636	4.00E-55	82.55
PREDICTED: ADP-ribosylation factor 6	*Tribolium castaneum*	3182	4.00E-82	72.8
PREDICTED: nuclear factor NF-kappa-B p110 subunit isoform X1	*Tribolium castaneum*	3301	3.00E-78	68.03
PREDICTED: cdc42 homolog	*Tribolium castaneum*	1786	3.00E-138	78.99
PREDICTED: glutamate [NMDA] receptor subunit 1 isoform X2	*Tribolium castaneum*	2932	0	71.43
PREDICTED: 1-phosphatidylinositol 4,5-bisphosphate	*Tribolium castaneum*	4244	0	69.03

phosphodiesterase gamma-1				
PREDICTED: SHC-transforming protein 1	*Tribolium castaneum*	5140	4.00E-130	69.36
PREDICTED: zinc finger protein 2 homolog isoform X1	*Tribolium castaneum*	4028	2.00E-67	74.49
PREDICTED: group XIIA secretory phospholipase A2	*Aethina tumida*	1472	2.00E-85	71.66
PREDICTED: ralA-binding protein 1	*Aethina tumida*	2419	1.00E-127	69.26
PREDICTED: embryonic polarity protein dorsal-like isoform X3	*Agrilus planipennis*	3037	3.00E-111	71.07
**Cell cycle**				
cell division cycle protein 16 homolog	*Anoplophora glabripennis*	1855	5.00E-139	75.56
cell division cycle protein 23 homolog	*Anoplophora glabripennis*	1849	0	73.85
cell division cycle protein 27 homolog	*Anoplophora glabripennis*	3090	5.00E-91	69.22
mitotic checkpoint serine/threonine-protein kinase BUB1 beta	*Anoplophora glabripennis*	3658	2.00E-98	73.21
mitotic spindle assembly checkpoint protein MAD1-like	*Anoplophora glabripennis*	2604	3.00E-24	64.24
14-3-3 protein epsilon	*Zootermopsis nevadensis*	1038	7.00E-146	75.53
14-3-3 protein zeta isoform X1	*Anoplophora glabripennis*	2161	0	83.52
PREDICTED: 14-3-3 protein zeta isoform X3	*Dendroctonus ponderosae*	1175	2.00E-68	80.07

anaphase-promoting complex subunit 4	*Anoplophora glabripennis*	2666	5.00E-53	65.92
anaphase-promoting complex subunit 10	*Leptinotarsa decemlineata*	1072	2.00E-137	80.87
PREDICTED: anaphase-promoting complex subunit 2	*Tribolium castaneum*	3131	2.00E-169	68.43
PREDICTED: cell division cycle protein 16 homolog	*Aethina tumida*	689	2.00E-37	71.72
structural maintenance of chromosomes protein 3	*Anoplophora glabripennis*	3851	0	75.59
PREDICTED: structural maintenance of chromosomes protein 1A	*Tribolium castaneum*	4067	0	74.69
dual specificity protein phosphatase CDC14B-like	*Anoplophora glabripennis*	2133	4.00E-116	69.17
histone deacetylase Rpd3 isoform X1	*Anoplophora glabripennis*	1908	0	78.01
transcriptional regulator Myc-1 isoform X2	*Anoplophora glabripennis*	3653	1.00E-24	74.18
probable ATP-dependent RNA helicase DDX27	*Anoplophora glabripennis*	2782	0	70.33
protein cortex-like	*Anoplophora glabripennis*	1877	0.11	95.83
growth arrest and DNA damage-inducible protein GADD45 alpha	*Leptinotarsa decemlineata*	1125	2.00E-31	70.63
RING-box protein 1A	*Onthophagus taurus*	588	6.00E-89	81.16
PREDICTED: G1/S-specific cyclin-D3-like	*Aethina tumida*	1476	3.00E-44	68
PREDICTED: cyclin-dependent kinase 2-like	*Aethina tumida*	1454	2.00E-141	72.06

PREDICTED: S-phase kinase-associated protein 1	*Agrilus planipennis*	305	6.00E-51	78.81
PREDICTED: S-phase kinase-associated protein 1 isoform X1	*Ceratosolen solmsi marchali*	845	3.00E-27	66.59
PREDICTED: cullin-1	*Tribolium castaneum*	3231	0	74.24
**Insect hormone biosynthesis**				
testis-specific aldehyde dehydrogenase, mitochondrial-like	*Mizuhopecten yessoensis*	331	2.00E-41	71.3
testis-specific PREDICTED: cytochrome P450 307a1-like	*Agrilus planipennis*	465	1.00E-22	69.93

Table S2. The C_t values of 29 tested unigenes for RT-qPCR analysis

Gene	Testis (C_t ± SE)	Ovary (C_t ± SE)
Testis-up-regulated unigenes		
TRINITY_DN36203_c0_g1	26.9213 ± 0.0269	--
TRINITY_DN36769_c0_g1	28.7448 ± 0.2658	--
TRINITY_DN32154_c0_g1	27.8281 ± 0.1527	--
TRINITY_DN34503_c0_g1	27.4787 ± 0.2203	--
TRINITY_DN3474_c0_g1	27.3564 ± 0.0576	--
TRINITY_DN34469_c0_g1	26.2939 ± 0.2123	--
TRINITY_DN38009_c0_g1	27.3262 ± 0.1442	--
TRINITY_DN37061_c0_g1	27.3928 ± 0.1300	--
TRINITY_DN38116_c0_g1	24.1200 ± 0.0146	--
TRINITY_DN39900_c0_g1	27.7148 ± 0.1271	--
TRINITY_DN36997_c0_g1	31.0963 ± 0.1055	--
TRINITY_DN36816_c1_g1	29.6950 ± 0.1461	--
TRINITY_DN40076_c0_g1	29.1139 ± 0.1323	--
TRINITY_DN37352_c0_g2	26.2067 ± 0.0948	--
TRINITY_DN28048_c0_g1	22.8569 ± 0.0961	37.2913 ± 0.1161
TRINITY_DN37903_c0_g1	25.0676 ± 0.1302	--
Ovary-up-regulated unigenes		
TRINITY_DN35620_c0_g2	--	27.7807 ± 0.2636
TRINITY_DN37242_c1_g2	--	30.9480 ± 0.3723
TRINITY_DN37127_c1_g1	--	31.0885 ± 0.1871
TRINITY_DN31659_c0_g1	--	30.9607 ± 0.2123
TRINITY_DN33161_c0_g1	--	30.2988 ± 0.2153

TRINITY_DN35988_c5_g2	--	31.1437 ± 0.1396
TRINITY_DN36847_c0_g3	37.3610 ± 0.2944	30.2949 ± 0.3812
TRINITY_DN32419_c0_g1	--	29.9618 ± 0.3918
TRINITY_DN37464_c0_g3	--	30.4087 ± 0.2826
TRINITY_DN38884_c0_g1	--	29.4363 ± 0.1464
TRINITY_DN24326_c0_g1	--	30.2033 ± 0.2681
TRINITY_DN40971_c0_g1	--	30.8122 ± 0.3285
TRINITY_DN34758_c0_g1	--	29.0019 ± 0.1400
RPL13	24.6785 ± 0.3439	23.2646 ± 0.0612

'—' cannot detect

Table S3. Primers used in semi-RT-qPCR and RT-qPCR for validation of differentially expressed genes.

Gene	Primer sequences (5′-3′)	Length (bp)	FC (O/T)	Log2FC (O/T)	Annotation
Testis-up-regulated unigenes					
TRINITY_DN36203_c0_g1-F	GAGCGTCGGTACACATAAA	131	0.00	-15.66	arginine kinase
TRINITY_DN36203_c0_g1-R	GTACACGCCTTCTTCAGAC				
TRINITY_DN36769_c0_g1-F	GTTTGGTCTCTTGGAGTTGT	126	0.00	-15.62	testis-specific serine/threonine-protein kinase 3
TRINITY_DN36769_c0_g1-R	CGTTACACTCGACCTGAAAG				
TRINITY_DN32154_c0_g1-F	CACAAGGAGGGAGCATTTAT	112	0.00	-15.28	L-lactate dehydrogenase isoform X2
TRINITY_DN32154_c0_g1-R	TCAGTGATTCCGTTTGATCC				

TRINITY_DN34503_c0_g1-F	CACCTTACCTCCTGACGATA	118	0.00	-15.17	venom allergen 5-like
TRINITY_DN34503_c0_g1-R	ATGCCACAGTCTCTCATTTC				
TRINITY_DN3474_c0_g1-F	GCTCTACTTGGTGCCTAATC	144	0.00	-15.13	alpha-1,3/1,6-mannosyltransferase
TRINITY_DN3474_c0_g1-R	TCCACAAGAGGCATCATAAC				ALG2-like
TRINITY_DN34469_c0_g1-F	CCTCGAAAGCCTATCATTCC	124	0.00	-15.09	glandular kallikrein-3,
TRINITY_DN34469_c0_g1-R	CAGGTTGTGGTAGTGGATTT				submandibular-like
TRINITY_DN38009_c0_g1-F	TCCCAACTCAGACTGTTCTA	104	0.00	-15.08	proteasomal ubiquitin receptor
TRINITY_DN38009_c0_g1-R	GTGGACGTGTTTGGACTATT				ADRM1-like
TRINITY_DN37061_c0_g1-F	CAGGGACAAACAGACTTACC	100	0.00	-15.08	hypothetical protein AMK59
TRINITY_DN37061_c0_g1-R	ACTCCTCTACAGTGGCTATT				
TRINITY_DN38116_c0_g1-F	CAACTGCTTCCGGTACTAAA	137	0.00	-14.76	parkin coregulated gene protein
TRINITY_DN38116_c0_g1-R	CCTCCTGTTCTTTCCATCAA				homolog
TRINITY_DN39900_c0_g1-F	CGCTAAGAGCTGAGGTACTA	128	0.00	-14.70	protein-glutamate O-methyltransferase
TRINITY_DN39900_c0_g1-R	TTGAAGCATTGTTGGACGA				
TRINITY_DN36997_c0_g1-F	CGAGTACACTGCCAAACTT	104	0.00	-14.62	carnitine O-acetyltransferase-like
TRINITY_DN36997_c0_g1-R	CCATGTCCAACGGGTTATT				
TRINITY_DN36816_c1_g1-F	CCTCATTGGTGCATCTACTC	131	0.00	-14.59	NADP-dependent malic enzyme-like
TRINITY_DN36816_c1_g1-R	CCTGTGGTGTACATTCAGTT				
TRINITY_DN40076_c0_g1-F	GCATTCTCGCCTGGTATTT	107	0.00	-14.57	NADP-dependent malic enzyme-like
TRINITY_DN40076_c0_g1-R	GCGTCTTCTGCAGTACATT				
TRINITY_DN37352_c0_g2-F	CATCGGAGGAAAGCTGTATG	123	0.00	-14.53	cytosol aminopeptidase-like
TRINITY_DN37352_c0_g2-R	CCCAAACCTGCTACTGAAAT				
TRINITY_DN28048_c0_g1-F	ACAGTGTTGCGAGATGAAA	118	0.00	-14.44	protein D3-like
TRINITY_DN28048_c0_g1-R	AGCCTTGGGTACTCCTAAA				
TRINITY_DN37903_c0_g1-F	GAAGATGCAGCGAACAATTAC	137	0.00	-14.42	tubulin alpha-4 chain-like
TRINITY_DN37903_c0_g1-R	CCAAAGGAGTGGAAGACAAA				

Ovary-up-regulated unigenes					
TRINITY_DN35620_c0_g2-F	GCGAATGGAACGGAGAAT	134	2779.18	11.44	uncharacterized protein LOC108911198
TRINITY_DN35620_c0_g2-R	TCGGACACTCGTGAAGAA				
TRINITY_DN37242_c1_g2-F	AGGATTCGTGAGAACACAAC	121	2047.99	11.00	uncharacterized protein LOC109609123
TRINITY_DN37242_c1_g2-R	GTCGACAACGGTTACCTTAC				
TRINITY_DN37127_c1_g1-F	TGTGGTTTGTGTGAAGTAGG	115	1725.60	10.75	uncharacterized protein LOC111513761
TRINITY_DN37127_c1_g1-R	CCTTTAGCAGGTCGTTCATT				
TRINITY_DN31659_c0_g1-F	GACACACAGCTATGGTGAAG	123	1584.11	10.63	Pxs
TRINITY_DN31659_c0_g1-R	CGATTCTTAGGTTCCGTTGG				
TRINITY_DN33161_c0_g1-F	GCTCGCTGTGGTGATATAAA	140	1473.29	10.52	nuclear cap-binding protein subunit 2
TRINITY_DN33161_c0_g1-R	TCAAGATTCGTGCCGTTAAT				
TRINITY_DN35988_c5_g2-F	GTACCGCCTACCATTGTAAC	134	1469.59	10.52	ankyrin-1-like
TRINITY_DN35988_c5_g2-R	GGAACCCTCAGCTTAACTTC				
TRINITY_DN36847_c0_g3-F	CAGTTTGTCACGATGCCTAT	121	1358.69	10.41	ceramide-1-phosphate transfer protein
TRINITY_DN36847_c0_g3-R	CAAACCTTTGCCAACAACTC				
TRINITY_DN32419_c0_g1-F	CAGACTTCGACCACTTTCAT	110	1293.37	10.34	ras-like GTP-binding protein RHO
TRINITY_DN32419_c0_g1-R	TTACTTCAGGGAGCCATTTC				
TRINITY_DN37464_c0_g3-F	GCAACTCGGAATCTTGGTAG	143	1221.33	10.25	REST corepressor 2-like
TRINITY_DN37464_c0_g3-R	ACACAGTCCTCCCTCTTTAT				
TRINITY_DN38884_c0_g1-F	GATTTGCCGCCAATACAAAT	128	1219.42	10.25	UDP-glucuronosyltransferase 1-7
TRINITY_DN38884_c0_g1-R	CTGTCCTGCTATGTGCTAAA				
TRINITY_DN24326_c0_g1-F	CGGACGATGAAGGAGATAGA	111	1149.35	10.17	uncharacterized protein LOC109609449
TRINITY_DN24326_c0_g1-R	CCGATGGACTTGGAGATGA				
TRINITY_DN40971_c0_g1-F	ACGGTATGATGACCCACA	117	1001.21	9.97	zinc finger protein 107-like
TRINITY_DN40971_c0_g1-R	GTCACGTACTGCCTCTCT				
TRINITY_DN34758_c0_g1-F	CATCCCAGAAGAACCTGAAA	128	812.59	9.67	protein snail homolog Sna-like

TRINITY_DN34758_c0_g1-R	ATGAGGGTGGAGGAGAAATA

O/T: Ovary/Testis

Table S4. Primers used for dsRNA synthesize

Gene	Primer sequences (5′-3′)	Length (bp)
dsRC2-like-F	taatacgactcactatagggATGAAGACCCGGCTGAAAC	394
dsRC2-like-R	taatacgactcactatagggAGCTCGCTTATTCGGTCAAA	
dsPSHS-like-F	taatacgactcactatagggGGTGTATTATCCCCGAGCAA	398
dsPSHS-like-R	taatacgactcactatagggGGTGTCTGTTTCCCTGTCGT	
